# Effect of task difficulty on blood-oxygen-level-dependent signal: A functional magnetic resonance imaging study in a motion discrimination task

**DOI:** 10.1371/journal.pone.0199440

**Published:** 2018-06-25

**Authors:** Ren Na, Taiyong Bi, Bosco S. Tjan, Zili Liu, Fang Fang

**Affiliations:** 1 School of Psychological and Cognitive Sciences and Beijing Key Laboratory of Behavior and Mental Health, Beijing, China; 2 Key Laboratory of Machine Perception (Ministry of Education), Beijing, China; 3 Peking-Tsinghua Center for Life Sciences, Beijing, China; 4 School of Management, Zunyi Medical University, Guizhou, China; 5 Department of Psychology, University of Southern California, Los Angeles, CA, United States of America; 6 Department of Psychology, UCLA, Los Angeles, CA, United States of America; 7 PKU-IDG/McGovern Institute for Brain Research Peking University, Beijing, China; University of Montreal, CANADA

## Abstract

There is much evidence that neural activity in the human brain is modulated by task difficulty, particularly in visual, frontal, and parietal cortices. However, some basic psychophysical tasks in visual perception do not give rise to this expected effect, at least not in the visual cortex. In the current study, we used functional magnetic resonance imaging (fMRI) to record brain activity while systematically manipulating task difficulty in a motion discrimination task, by varying the angular difference between the motion direction of random dots and a reference direction. We used both a blocked and an event-related design, and presented stimuli in both central and peripheral vision. The behavioral psychometric function, across angular differences of 3°, 9°, 15°, or 80°, spanned the full response range, as expected. The mean blood oxygen level dependent (BOLD) signals were also correlated within-participants between the blocked and event-related designs, across all brain areas tested. Within the visual cortex, the voxel response patterns correlated more within-conditions (e.g., 3° and 3°) than between-conditions (e.g., 3° and 9°), in both designs, further attesting to the reasonable quality of the BOLD data. Nevertheless, the BOLD-o-metric functions (i.e., BOLD activity as a function of task difficulty) were flat in the whole-brain and region-of-interest (ROI) analyses, including in the visual cortex, the parietal cortex, in both designs, and in foveal and peripheral visual fields alike. Indeed, there was little difference between BOLD activity during the 3° and 80° conditions. Some suggestive evidence of difficulty modulation was revealed only in the superior and inferior frontal gyri for the blocked design. We conclude that, in motion discrimination, there is no systematic BOLD modulation that accompanies the standard psychometric function across different hierarchies of cortical areas, except for the frontal lobe of the brain.

## 1. Introduction

Task difficulty has been found to qualitatively change the way the brain functions, according to the existing literature. The reverse hierarchy theory of visual perceptual learning, proposed by Ahissar, Hochstein [1], suggests that different visual hierarchies, involved with the learning of easy tasks and difficult tasks, show generalization and specificity, respectively. Other perceptual learning studies provided further evidence of a difficulty effect by showing different learning and transfer effects for easy and difficult tasks, across multiple task types such as motion discrimination [2,3] and orientation discrimination [4]. These behavioral effects suggested that brain activity was likely also modulated by task difficulty and raised an important question concerning the neural processing of visual stimuli.

Brain imaging studies have shown that task difficulty affects brain activity in a variety of tasks. fMRI studies have suggested that, in general, task difficulty changes the BOLD responses mostly in frontal and parietal cortices. For example, in a working memory task, activity in the anterior cingulate cortex (ACC) and other regions of the prefrontal cortex (PFC) was found to increase with increasing task difficulty, manipulated by adjusting the degree of stimulus degradation [5]. Attentional load related difficulty in a visual tracking task was also found to affect the activation in the parietal and frontal cortical areas, including the superior frontal sulcus (SFS) [6]. Other studies concerning the effect of task difficulty on neural responses consistently demonstrated that the responses of frontal, parietal, and cingulate cortices were affected by task difficulty, across a variety of cognitive tasks including working memory [5,7,8], language [9–11], time perception [12,13], and visual attention [14,15]. Generally speaking, the frontal lobe was recruited in diverse cognitive demands and thus showed sensitivity to task difficulty [16].

In addition to fMRI studies, electroencephalograph (EEG) studies showed that when an oddball task became more difficult (as the sizes of target and distractor stimuli became more similar), the distractor P3a amplitude increased while the target P3b amplitude decreased [17]. The generators of these P3 components were suggested to lie bilaterally in the frontal, parietal, and temporal areas using fMRI-constrained source analyses [18]. Besides P3a and P3b, the amplitude of another late component termed D220 was found directly proportional to the difficulty levels of decision making [19], which indicated that difficulty might influence the late stage of visual processing. Furthermore, a Positron Emission Tomography (PET) study in 107 subjects also showed that significant increases in cerebral blood flow (CBF) in ACC were more likely to occur during difficult tasks [20]. Converging evidence, therefore, demonstrates the close relationship between task difficulty and neural activity in higher-level cortical areas such as the frontal, parietal, and cingulate cortices, in multiple tasks.

Previous studies investigating task difficulty focused primarily on the frontal and parietal cortices, outside the sensory cortex. The current evidence as to whether task difficulty modulates the activity of lower-level visual cortices, however, is less consistent. A single-cell recording study in rhesus monkey V4 neurons showed that neuronal responses to stimuli became larger and more selective when the visual discrimination task was more difficult [21]. However, ultimately the increasing amount of attention, rather than discrimination difficulty, was found to enhance the responsiveness and selectivity of the neurons processing the stimulus. The influence of task difficulty on rhesus monkey V1 neurons was also modulated by spatial attention, with an increase in firing rate at the focus of attention and a suppression in surrounding regions [22]. In addition to neurophysiological studies, fMRI results showed that the base responses in V1, V2, and V3 depended on task difficulty, with activity decreasing as the pattern-detection task became easier [23]. However, several other fMRI studies suggested that task difficulty did not change the BOLD responses. Sunaert et al. [24] found that increasing the difficulty of a motion speed discrimination task had little effect on the BOLD responses in visual areas. Only dorsal V3 showed a weak difficulty effect. Furthermore, Ganis et al. [25] found that no activation in the brain could be predicted by the response time (an indicator of task difficulty) of visual imagery and visual perception tasks. Other fMRI studies, using ROI analyses, also showed a null effect of task difficulty on the activity in the human visual cortex. For example, manipulation of the angular size in an orientation discrimination task gave rise to flat BOLD responses in V1, V2, V3a, V3b, and V4 [26,27]. Taking these findings together, there is still no consensus about the effect of task difficulty on the neural responses in the visual cortex.

At present, brain imaging studies mainly rely on univariate analysis and multivariate pattern analysis (MVPA) to quantify the effect of some variable on brain activity. In visual discrimination tasks, difficulty is often increased by decreasing the signal-to-noise ratio of the stimuli, thus requiring more effort and more attentional resources from the participant. As a result, greater brain activation is expected to take place as measured by the fMRI BOLD peak. The current study was designed to test whether there is a systematic univariate change in brain activity as a function of the difficulty of a motion discrimination task, as defined by the angular difference in motion direction discrimination. Note that the meaning of task difficulty varies greatly, depending on how it is defined in a specific task. The motion discrimination difficulty has never been studied by fMRI previously. Therefore, although we would expect a similar modulation effect in the frontal and parietal cortex, such an effect is not necessarily guaranteed, since no existing evidence supports the difficulty effect per our definition of difficulty.

The difficulty effect found in previous studies may result from the ways in which they manipulated task difficulty. These manipulations may not only vary the difficulty levels, but also introduce confounding factors such as different perceptual loads or different physical properties of stimuli (e.g., contrast, coherence level in dot motion). In addition, previous studies testing the effect of task difficulty may have focused only on the visual cortex [26,27]. Furthermore, it is not known how the trial-structure of an experiment (e.g., blocked versus event-related design) affects attentional load. More specifically, in a blocked design, the attentional demand may be different between an easy and a difficult block. In an event-related design, however, since the difficulty level of each trial is unpredictable, the attentional demand is expected to be approximately equal between easy and difficult trials. In the current study, we adopted a motion direction discrimination task, where task difficulty was manipulated by changing the directional difference between a motion stimulus and a reference. This manipulation kept the physical stimuli nearly the same between different difficulty levels. Using fMRI, we measured brain activity across visual cortical areas in both the ventral and dorsal visual streams, and in multiple higher-order cortices. We also performed a whole-brain analysis to search for difficulty-level-related areas across the entire brain. In addition to these univariate analyses, we also performed MVPA to investigate the activity patterns in the visual cortex at different difficulty levels. We presented stimuli both in the fovea and in the periphery (in a supplementary experiment), to further inspect the difference between foveal and peripheral visual processing. An additional series of analyses, including applying spatial smoothing to fMRI data, comparing between the easiest and most difficult conditions directly, conducting denoising methods to improve fMRI data, as well as selecting the best participants and better ROIs, were implemented for further investigation into our data (see Supplemental Material 5).

Our results showed that even though the activity patterns in the visual cortex changed with different difficulty levels in the MVPA analysis, the brain activity as measured by univariate BOLD signals was invariant to task difficulty across different hierarchies of cortical areas, except for some superior and inferior frontal areas in the blocked design. In the visual cortex, the null effect of task difficulty was robust for both blocked and event-related designs, and in both foveal and peripheral visual fields.

## 2. Experimental procedures and results

### 2.1. Experimental procedures

**Fig 1:**
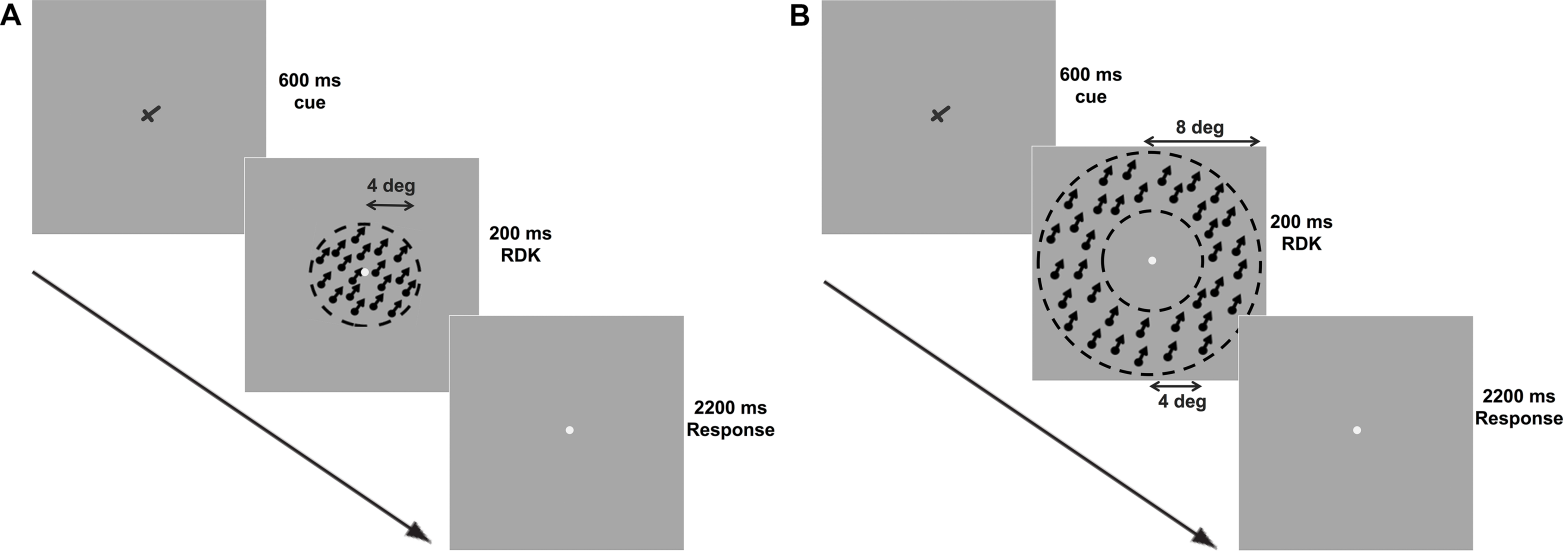
Stimulus and schematic description of a trial. (A) Experiments 1 and 2: In each 3 sec trial, a cross was first presented at the center of the screen for 600ms. Its longer arm indicates the reference direction. After this reference, an RDK motion stimulus in a circular region with a radius of 4° was presented for 200ms. Participants discriminated whether the motion direction was clockwise or counterclockwise of the reference. The motion directions of RDK were 3°, 9°, 15° from the reference in Experiment 1, and 3°, 9°, 15°, 80° from the reference in Experiment 2, corresponding to the three and four levels of difficulty. (B) Supplementary Experiment: The moving dots of RDK were restricted to an annular region with an outer radius of 8° and an inner radius of 4°. The stimuli and experimental design were otherwise the same as in Experiment 2.

During fMRI scanning, 24 participants (14 for Experiment 1, 10 for Experiment 2; 9 males) performed a motion direction discrimination task, in which they judged whether the motion direction of a random-dot kinematogram (RDK) stimulus was clock- or counter-clockwise relative to a static reference direction (Fig 1A). Four more participants performed a Supplementary Experiment, in which the RDK was presented in an annular region in the peripheral visual field, instead of the foveal visual field (Fig 1B). The task difficulty was manipulated by changing the angular difference between the reference and RDK directions (3°, 9°, and 15° in Experiment 1; 3°, 9°, 15°, and 80° in Experiment 2 and Supplementary Experiment). Both blocked and event-related designs were applied in all three experiments. All participants gave written, informed consent in accordance with the procedures and protocols approved by the human subject review committee of Peking University, Beijing, China.

### 2.2. Behavioral results

**Fig 2:**
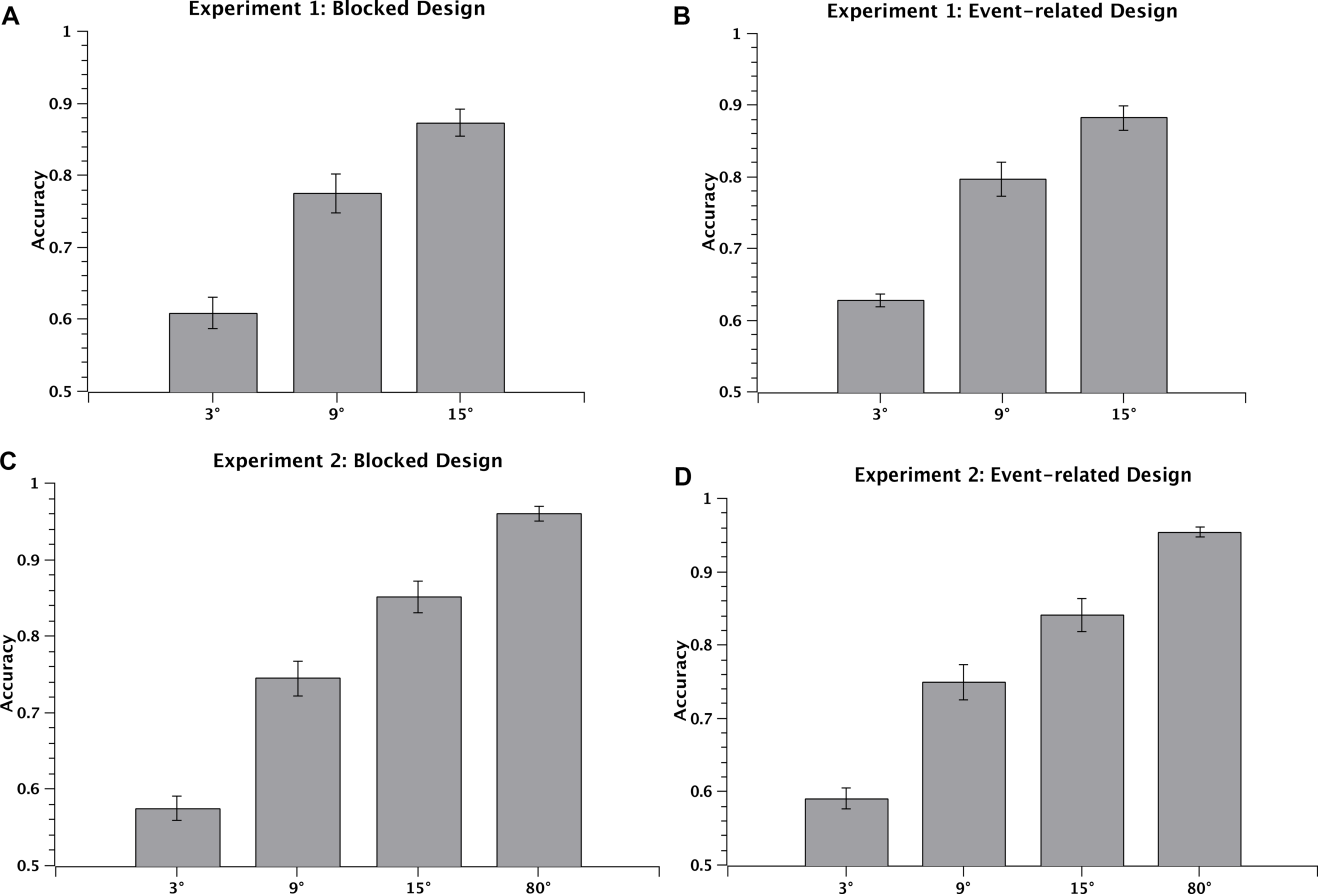
Behavioral results: Experiments 1 and 2. Behavioral accuracies averaged across 14 participants in 3°, 9°, and 15° conditions for the blocked (A) and event-related (B) designs in Experiment 1; and across 10 participants in 3°, 9°, 15°, and 80° conditions for the blocked (C) and event-related (D) designs in Experiment 2. Error bars denote 1 SEM of the mean here and in all subsequent figures.

In Experiment 1, we measured participants’ accuracy (percent correct choices) across the three difficulty levels (3°, 9°, and 15°), separately for the blocked and event-related designs. The accuracies averaged across all participants for the 3°, 9°, 15° conditions were 60.9%, 77.4%, 87.3%, respectively, for the blocked design (repeated-measures ANOVA, F(2, 26) = 114.9, p < 0.001; post-hoc paired t-test, all t(13) > 7.8, p < 0.001) (Fig 2A); and 62.8%, 79.7%, 88.2% for the event-related design (repeated-measures ANOVA, F(2, 26) = 117.0, p < 0.001; post-hoc paired t-test, all t(13) > 6.2, p < 0.001) (Fig 2B). In Experiment 2 there were four difficulty levels (3°, 9°, 15°, and 80°) for both blocked and event-related designs. The accuracies averaged across all participants for the 3°, 9°, 15°, 80° conditions were 57.5%, 74.5%, 85.1%, 96.0% for the blocked design (repeated-measures ANOVA, F(3, 21) = 121.2, p < 0.001; post-hoc paired t-test, all t(7) > 5.3, p < 0.001) (Fig 2C); and 59.1%, 74.9%, 84.1%, 95.4% for the event-related design (repeated-measures ANOVA, F(3, 21) = 124.4, p < 0.001; post-hoc paired t-test, all t(7) > 5.1, p < 0.001) (Fig 2D).

Behavioral results from Experiments 1 and 2 consistently showed that performance for both blocked and event-related designs significantly improved as the angular difference increased. Behavioral results from the Supplementary Experiment followed the same pattern, as shown in supporting information (S1 Fig).

### 2.3. MRI data analysis and data quality

**Fig 3:**
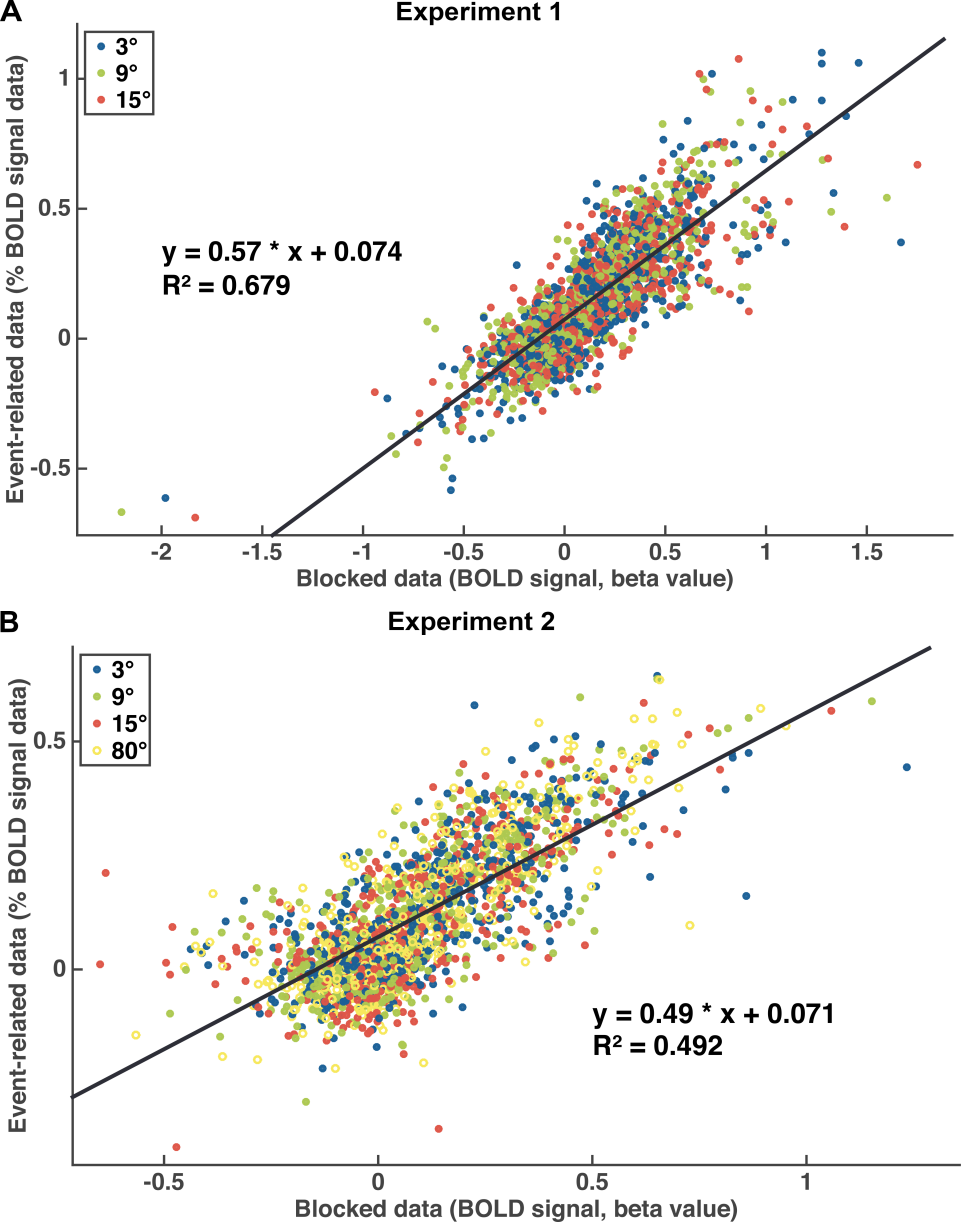
Correlation between blocked data and event-related data. Pearson correlation between beta values for the blocked design and response peaks for the event-related design in Experiment 1 (A) and in Experiment 2 (B) (blue: 3°, green: 9°, red: 15°, hollow yellow circle: 80°).

We defined visual cortex ROIs in V1, V2, V3, V3a, V4, MT+, and IPS; and 42 spherical ROIs in the higher-order cortex, using the method specified in Supplemental Material 4. For the blocked design, we extracted BOLD signals by averaging the data across voxels in each pre-defined ROI, and then used a general linear model (GLM) to estimate the beta value for each ROI and each task difficulty condition. For the event-related design, we again averaged BOLD signals within each ROI, then normalized to average signal intensity and the base-line response was subtracted from the response to test stimuli. Finally, the peak of the response time course was obtained for each ROI and each condition, as a measure of activation level. In order to examine the consistency of our results between the blocked design and event-related design, we calculated the Pearson correlation between the beta values from the blocked design and the response peak magnitudes from the event-related design, across all the ROIs, participants, and difficulty conditions, separately for Experiments 1 and 2. As shown in Fig 3, the BOLD activity was similar between the blocked and event-related designs (r^2^ = 0.68 in Experiment 1, and 0.49 in Experiment 2), demonstrating the robustness and stability of MRI data across the two experimental designs.

### 2.4. Difficulty of motion direction discrimination task revealed in the visual cortex with MVPA

**Fig 4:**
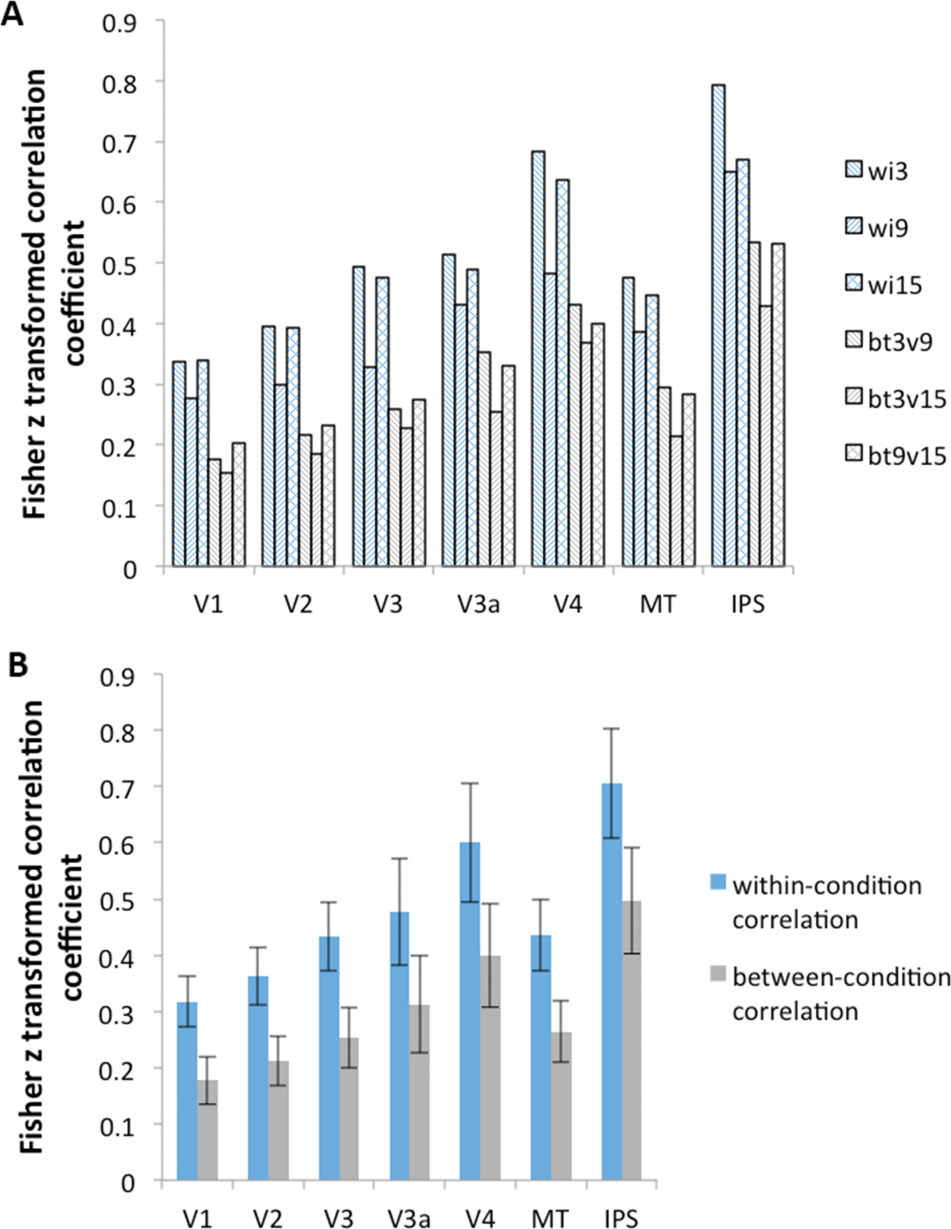
MVPA results. The correlation between activity patterns of motion stimuli within the same condition was larger than that between different conditions in Experiment 1 (A) and in Experiment 2 (B).

To account for the possibility that task difficulty is represented by a more complex code within an ROI than can be detected with univariate methods, we implemented an additional MVPA analysis on the blocked design data. Specifically, we correlated the multi-voxel activity patterns between different blocks, either of the same difficulty level or of different difficulty levels. If the neural representations of motion stimuli are changed by task difficulty, then the correlation between activity patterns in blocks of different difficulty levels (between-condition correlation) should be smaller than the correlation between activity patterns in blocks of the same difficulty level (within-condition correlation). Otherwise, if difficulty does not modulate multi-variate BOLD activity, there should be no significant difference between the within-condition correlation and between-condition correlation. This analysis was run separately on each of the seven visual ROIs described earlier, to determine which ROIs, if any, were modulated by task difficulty. The MVPA results showed that in all seven visual cortical ROIs, the within-condition correlations were significantly greater than between-condition correlations in Experiment 1 (all paired t > 4.5, p < 0.001) (Fig 4A) and in Experiment 2 (all paired t > 8.1, p < 0.001) (Fig 4B), suggesting that difficulty level is represented in the neural activity of these brain regions.

### 2.5. Univariate ROI analysis as a function of task difficulty

**Fig 5:**
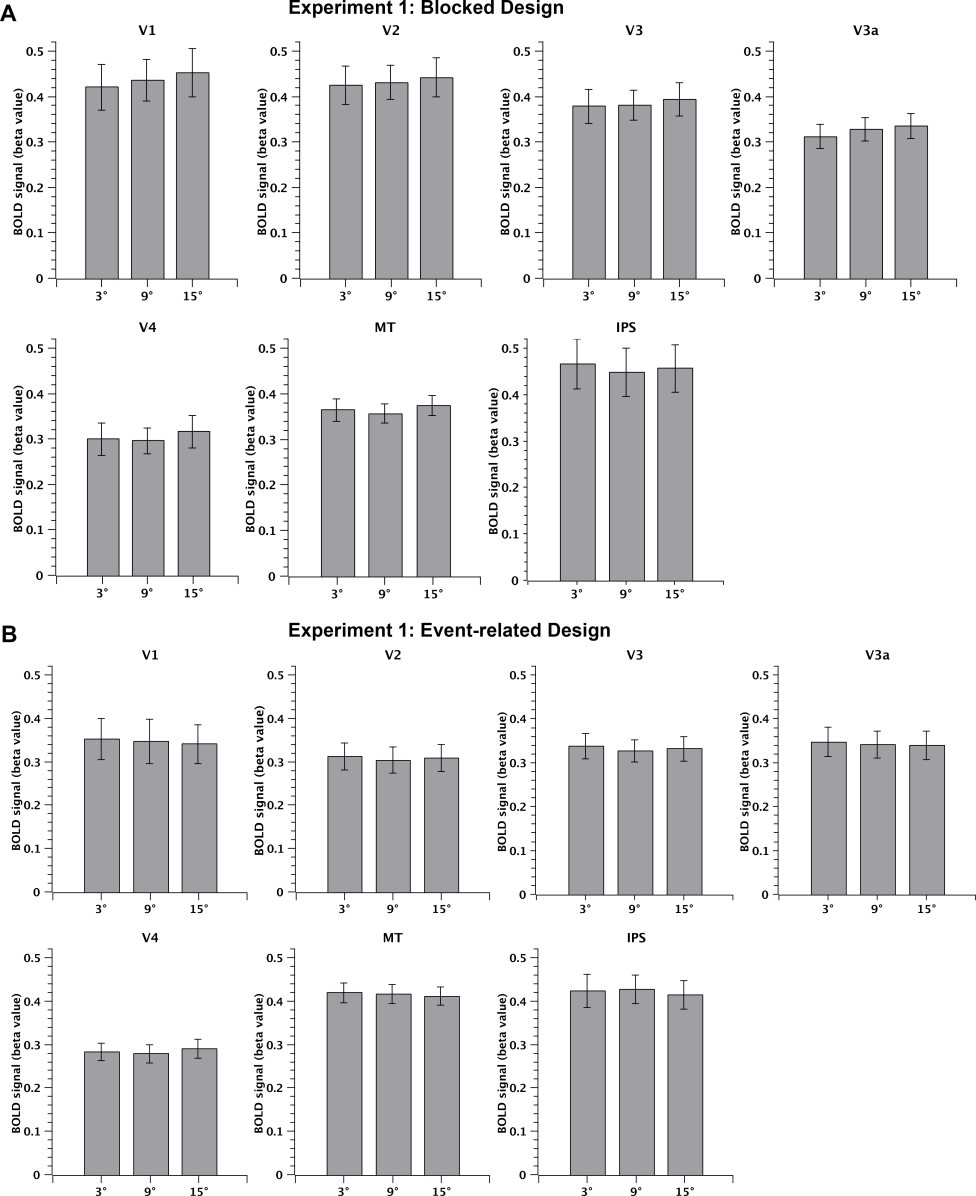
MRI results of ROI analysis: Experiment 1. BOLD amplitudes averaged across 14 participants for the 3°, 9°, and 15° conditions in V1, V2, V3, V3a, V4, MT+, and IPS for the blocked design (A) and the event-related design (B).

**Fig 6:**
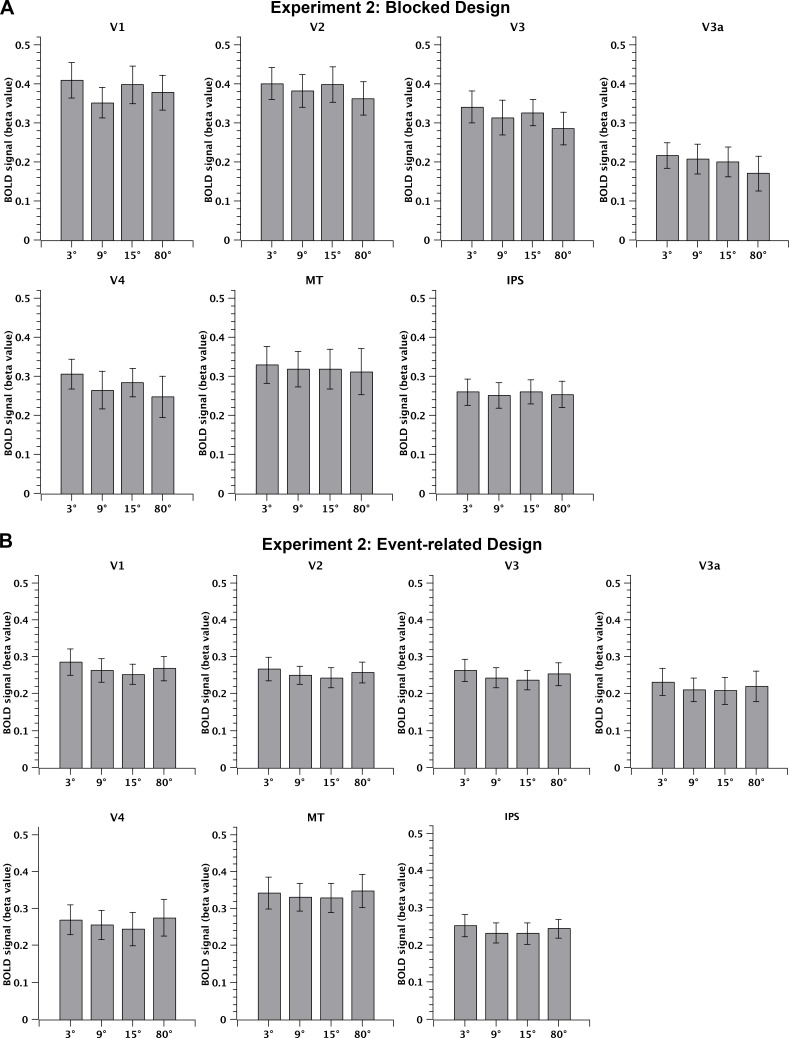
MRI results of ROI analysis: Experiment 2. BOLD amplitudes averaged across 10 participants for the 3°, 9°, 15°, and 80° conditions in V1, V2, V3, V3a, V4, MT+, and IPS for the blocked design (A) and the event-related design (B).

As described in section 2.3, we obtained BOLD amplitudes at each of seven visual cortical ROIs and during each condition (difficulty level; 3°, 9°, and 15° in Experiment 1; and 3°, 9°, 15°, and 80° in Experiment 2), for both the blocked design (beta values estimated with a GLM) and event-related design (peak response of the time course measured as the signal percentage change). A repeated-measures ANOVA on BOLD amplitudes was performed with task difficulty as the within-participant factor in each ROI, and for both the blocked and event-related designs. In Experiment 1, the main effect of difficulty was not significant in any ROI for either design, i.e., there was no significant difference in the activation strength among three levels of task difficulty (the blocked design: all F(2, 26) < 2.7, p > 0.05, Fig 5A; the event-related design: all F(2, 26) < 0.97, p > 0.05, Fig 5B). Similarly, in Experiment 2, the main effect of difficulty was not significant in any ROI (the blocked design: all F(3, 21) < 2.2, p > 0.05, Fig 6A; the event-related design: all F(3, 21) < 2.1, p > 0.05, Fig 6B). The time courses of the BOLD signal for the event-related design are shown in Supplemental Material 3. In sum, the univariate analyses indicated that difficulty of the motion direction discrimination task did not systematically influence average cortical activations in the visual cortical areas, in either the blocked or event-related designs.

For higher-order cortical ROIs, 42 spherical ROIs were defined based on Talairach coordinates that had been found to be modulated by task difficulty in previous literatures (Table 1). This same analysis was applied to all 42 ROIs for the blocked and event-related designs in Experiments 1 and 2. Again, no ROI displayed significant differences of BOLD amplitude across task difficulty conditions for either design (all p > 0.05, FDR corrected), suggesting that task difficulty did not affect activation strength in these regions either.

**Table 1:** The 42 Talairach coordinates for ROI analysis in the higher-order cortex.

ROI	Region	Hemisphere	Talairach coordinate			Reference
			x	y	z	
**1**	Precuneus	R	12	-65	51	Gould et al. [7]
**2**	Fusiform gyrus	L	-22	-66	-8	
**3**	Precuneus	R	4	-58	53	
**4**	Middle occipital gyrus	R	32	-85	1	
**5**	Cerebellum	L	-32	-53	-12	
**6**	Middle occipital gyrus	R	32	-83	15	
**7**	Anterior cingulate gyrus	L	-6	12	42	
**8**	Cerebellum	R	28	-55	-12	
**9**	Superior occipital gyrus	L	-30	-76	33	
**10**	Middle occipital gyrus	L	-40	-63	-10	
**11**	Anterior cingulate gyrus	L	-4	27	34	
**12**	Middle occipital gyrus	L	-46	-66	-7	
**13**	Superior frontal gyrus	L	-33	27	45	Sunaert et al. [24]
**14**	Anterior portion of insula	R	57	15	-3	
**15**	Lateral occipital cortex	R	45	-75	15	
**16**	Dorsal intraparietal sulcus	R	27	-60	51	
**17**	Inferior frontal gyrus	R	45	21	15	
**18**	Inferior frontal gyrus	L	-51	30	-3	
**19**	Superior frontal gyrus	L	-15	42	42	
**20**	Middle frontal gyrus	R	39	45	24	
**21**	Anterior cingulate cortex	-	-3	33	54	
**22**	Anterior cingulate cortex	-	0	45	39	
**23**	Superior parietal lobule	R	27	-63	54	Dräger et al. [10]
**24**	Precuneous	R	27	-72	51	
**25**	Inferior parietal lobule	R	42	-51	60	
**26**	Postcentral gyrus	R	45	-39	57	
**27**	DLPFC	L	-37	42	29	Barch et al. [5]
**28**	Inferior frontal cortex	L	-57	2	13	
**29**	Parietal cortex	L	-40	-50	50	
**30**	Basal ganglia	-	25	-1	9	
**31**			39	15	36	Duncan, Owen [16]
**32**			48	12	21	
**33**			-51	15	42	
**34**			0	30	45	
**35**			9	33	21	
**36**			-3	30	18	
**37**	Frontal lobe and precuneus		9	45	-9	
**38**			35	15	28	
**39**			4	25	43	
**40**			48	19	23	
**41**			50	19	2	
**42**			26	42	32	

We selected 42 Talairach coordinates in the higher-order cortex according to the existing results in previous articles concerning the task difficulty effect.

### 2.6. Univariate whole brain analysis as a function of task difficulty

Beyond our selected ROIs, we searched for any area in the entire brain that may show a task difficulty effect. We compared the BOLD signal at each voxel of the MNI (Montreal Neurological Institute) atlas brain between the different difficulty levels (3°, 9°, and 15° in Experiment 1; 3°, 9°, 15°, and 80° in Experiment 2), for both blocked and event-related designs. However, we did not find any voxels showing a significant difference between these conditions (p > 0.05, FDR corrected), across either experiment.

### 2.7. Correlation between behavioral and neural responses

In order to investigate the relationship between behavior (accuracy and response latency) and neural activity, we compared the BOLD signal during “correct” and “incorrect” trials, and separately correlated BOLD amplitude with behavioral response times. No significant difference in BOLD activity was found between “correct” and “incorrect” responses in any of the 49 ROIs (7 visual cortical ROIs and 42 higher-order cortical ROIs) (all p > 0.05, Bonferroni corrected), suggesting that these brain regions did not influence the accuracy of motion detection during the task. To evaluate the correlation between BOLD activity and behavioral response times, a Fisher-transformation was applied to the correlation coefficients to obtain z values. Only 3 out of 49 ROIs exhibited a correlation with z values being larger than 0.1 in both experiments (Experiment 1: z = 0.11 in IPS, z = 0.18 in ROI 1, z = 0.19 in ROI 7; Experiment 2: z = 0.11 in IPS, z = 0.12 in ROI 1, z = 0.17 in ROI 7). High correlations between behavioral response time and neural activity were found in IPS, ROI 1 (located at the right precuneus), and ROI 7 (located at the left anterior cingulate gyrus).

### 2.8. Supplementary experiment results

The behavioral and MRI results of the Supplementary Experiment, in which the stimuli were presented in the peripheral visual field, were consistent with Experiments 1 and 2 (S1 and S2 Figs). Despite the lack of statistical testing (due to the limited number of participants), the flat function of BOLD response against task difficulty in the Supplementary Experiment followed the same pattern as that in Experiments 1 and 2, in which the stimuli were presented in the foveal visual field. This serves as further support that task difficulty does not affect neural activity in the visual cortex, regardless of whether it was foveal or peripheral visual processing.

### 2.9. Additional analyses

In order to ensure that our null effects from the univariate analyses were not driven by particular parameters chosen in our analysis methods, we performed additional data analyses, including using spatially smoothed fMRI data, comparing 3° and 80° conditions directly, using fMRI data denoised by Kay et al. [28]’s method, as well as selecting the best participants’ data and better ROIs to analyze. The methods and results are in Supplemental Material 5.

We replicated our findings of null effect from the entire participant group when we re-ran our analyses using only the four best participants (based on behavioral performance and imaging data quality). An additional analysis using spatially smoothed data revealed only one ROI significantly modulated by task difficulty. However, given the weak activation level at this ROI, the evidence from this particular analysis appears not very strong. The ROI analysis using denoised data revealed a significant difficulty effect (BOLD response decreased as task difficulty decreased. Figs B and C in S1 File) at ROI 7, located at the left anterior cingulate gyrus, for only the event-related design. Finally, we combined data from Experiments 1 and 2 and performed ROI analysis across all 24 participants. ROI 30, which was located in the basal ganglia, was found to be significantly modulated by task difficulty (BOLD response increased as task difficulty decreased. S4 and S5 Figs) in the blocked design only. These significant results occurred in one of the two designs but not consistently across both, providing only some suggestive effects of task difficulty rather than a robust conclusion.

To avoid a possibly overly conservative criterion when a large number of candidate ROIs were used, we selected the six ROIs that showed difficulty modulation in Sunaert et al. [24]’s study. The task used by Sunaert et al. [24] was motion speed perception, which was most similar to our own task in the entire literature on brain imaging of task difficulty. Here, difficulty modulation was indeed found when data from Experiment 1 were analyzed, or when the combined data from both Experiments 1 and 2 were analyzed. Data from Experiment 2 alone did not yield statistical significance. This is suggestive evidence that task difficulty as defined in our motion direction task could modulate BOLD activation in the frontal lobe of the brain.

## 3. Discussion

### 3.1. A Flat BOLD response in the visual cortex against task difficulty in the fovea and periphery

In the current study, we investigated the effect of task difficulty (of motion direction discrimination) on BOLD activity across the entire brain. In Experiment 1, three levels of difficulty were adopted by manipulating the angular difference between the direction of a reference and a motion stimulus (RDK). Psychophysical results showed that the accuracy robustly increased as task difficulty decreased, across all participants. In contrast, our univariate fMRI results showed a flat BOLD response across task difficulty levels in seven pre-defined visual areas (V1, V2, V3, V3a, V4, MT+, and IPS), and for both event-related and blocked designs. In Experiment 2, we extended the range of task difficulty to an extremely easy level in which the angular difference in each trial was so large that little demand was required for the participants to perform this task. However, we still did not find any significant modulation of BOLD activity by difficulty level at any of the above seven visual ROIs, using the same univariate approach. In addition to the seven visual cortical areas, we also examined 42 spherical ROIs, defined manually based on previous literature. However, no significant difference in BOLD activity was found as a function of difficulty, at any of these higher-order cortical ROIs. Even when we examined the activation difference between 3° and 80° conditions directly, there was still no significant difference. The whole brain search similarly did not identify any voxels whose activity was significantly modulated by task difficulty. The null result was consistent across Experiments 1 and 2, and in both blocked and event-related designs.

Given the possibility that foveal visual processing might be different from peripheral visual processing, we performed a supplementary experiment, in which stimuli were presented only in the peripheral visual field, with a blank circular area in the center. Although the number of participants (four) in the Supplementary Experiment was not enough for statistical analysis, the activations in all the seven visual cortical ROIs showed the same tendency as in Experiments 1 and 2; namely, that there was no obvious difference in brain activity between the three or four task difficulty levels. These consistent activation patterns further supported the conclusion that task difficulty did not affect brain activity, regardless of the location of the visual field in which stimuli were presented.

### 3.2. Different multi-variate patterns across difficulty conditions

In addition to investigating the univariate BOLD activation levels at individual ROIs, as a function of task difficulty, we also explored the influence of task difficulty on neural response patterns using an MVPA method. MVPA of fMRI data is more sensitive and more informative about the functional organization of cortex than univariate analysis is with the GLM [29]. This method revealed that the multi-voxel patterns within each of the seven visual ROIs were more correlated within-condition (i.e., blocks of the same difficulty level) than between-condition (i.e., blocks of different difficulty levels), suggesting that difficulty affected the subtle neural patterns induced by motion stimuli. Considering the failure to find difficulty-related effects using univariate methods, we can assume that task difficulty (here manipulated by the angular difference in a motion direction discrimination task) modulated neural responses by changing the patterns of activity in the visual cortex, in contrast to simpler univariate measures that indicate the extent to which a cortical field or system is globally engaged [29].

### 3.3. Denoised data revealed the ACC and basal ganglia modulated by task difficulty

Some additional analyses were performed, including using spatially smoothed fMRI data and denoised data (Supplemental Material 5). However, after extensive effort to search for univariate BOLD activation modulation by task difficulty, the only brain areas we could find were the basal ganglia, which was found in previous literature to be a major structure for processing internal reward information [30–32]; and the anterior cingulate gyrus, which was found to be robustly correlated with task difficulty by a PET study across multiple tasks [20] and an fMRI study in a paired associates learning task [7]. Activity in the left anterior cingulate gyrus decreased as task difficulty decreased (Figs B and C in S1 File), suggesting that anterior cingulate gyrus activity is positively correlated with task difficulty. On the other hand, activity in the basal ganglion increased as task difficulty decreased (S4 and S5 Figs). This negative correlation may be because lower difficulty levels lead to higher behavioral accuracy, and thus higher reward, which may lead to higher neural responses in reward-related areas. These two ROIs were initially selected because they were demonstrated to be associated with task difficulty in previous literature [5,7]. Denoising the fMRI data helped reveal some suggestive effects caused by difficulty and internal rewards in higher-order cortices. Nevertheless, the findings were not robust as they were not consistent across two designs.

### 3.4. The frontal areas with suggestive difficulty effect implied default mode network activities

A suggestive task difficulty effect was revealed in some superior and inferior frontal areas when a limited number of ROIs were selected (see Supplemental Material 5). Their negative activities during task engagement, especially for the blocked design, implied their possible involvement in the default mode network (DMN) activities.

### 3.5. The IPS, precuneus, and ACC Activities were correlated with response times

High correlations between behavioral response time and neural activity in IPS, the right precuneus, and the left anterior cingulate gyrus suggest that these areas may influence the participants’ ability to discriminate the stimuli, and thus would be associated with the participants’ behavioral responses. This behavior-BOLD correlation is in accord with the Drift diffusion model of perceptual decision-making [33,34], which demonstrates a critical link between cognitive state and IPS activity [35,36]. The model posits that the IPS accumulates evidence from ventral temporal cortex over time and exhibits an activity level that is monotonically related to accumulation time. Although the neural activity was not correlated with task difficulty directly, it was significantly correlated with behavioral response time, found in previous literature and also supported by our results. This correlation might be due to the accumulation of activity at middle visual processing stages, instead of the task difficulty itself. On the other hand, behavioral accuracy was not significantly correlated with neural activation. Another fMRI study using modeling also demonstrated accumulator regions in the MT+, the IPS, and the inferior frontal sulcus that were modulated by task difficulty in a motion coherence task [37]. We can assume that changes in motion direction deviation might activate similar but not completely the same accumulators activated by stimulus strength (changes in motion coherence versus stimulus contrast).

### 3.6. Different designs elicited different results from previous studies

Our null effect of difficulty measured by univariate BOLD levels, in the visual cortex and most higher-order cortical areas found in the main analyses are in contradiction with some of the existing literature. A common assumption in previous studies was that participants might pay more attention to difficult stimuli and thus show a larger response in the visual cortex. Consistent with this assumption and introduced in the introduction, an electrophysiological study found that the response amplitude and selectivity of V4 neurons were both enhanced in difficult blocks compared to easy blocks [21]; an fMRI study found that V1 responses decreased as the task became easier [23]; another fMRI study discovered a significant correlation between neural activity in the frontal and parietal cortices and task difficulty in a visual tracking task [6]; some fusiform and occipital areas also showed a significant correlation with difficulty [8]. Note that these studies may not have independently manipulated task difficulty and attention on purpose. As a result, the effect of difficulty might be confounded with that of attention. Boudreau et al. [38] manipulated difficulty and attention separately in a detection task and found that the increase of difficulty only caused a modest increase in the response of V4 neurons. Other fMRI studies introduced in the introduction, in which attention was controlled carefully, also demonstrated that the activity of human early visual cortex and regions in the ventral stream were not affected by difficulty [24]; a whole brain search showed that activity in visual cortex could not be predicted by reaction times in perceptual tasks [25]. These pieces of evidence indicate that neural activity in the visual cortex might not be affected by task difficulty when attention is controlled properly, which is consistent with our results. The task design that we adopted in the current study focused on difficulty level itself, without changing participants’ attentional efforts purposely. In the event-related design experiment, participants were unable to predict the angular difference in each trial, and therefore they were probably not allocating their attention differently across different trials. If we find BOLD being modulated by task difficulty in the blocked design experiment, it might be attributed to the possibility that participants may have attended differently in anticipation of the block’s difficulty and thus there is a confound. In our case, we only found suggestive modulation for the blocked design in the negatively activated frontal lobe areas, when a targeted analysis was performed in some specific ROIs. Therefore, we could assume that if there is any anticipatory attention related modulation, its effect is weak enough across the task-activated brain areas such that no statistical significance could be found; while in the frontal lobe (likely part of the DMN) with negative activations, significant modulation was found as a function of the task difficulty in the blocked design experiment. Regarding the task-activated responses we acknowledge that the lack of an effect on difficulty does not mean that the effect does not exist. It is always possible that the difficulty effect existed but was too weak for us to find in our experimental paradigm. Nevertheless, we have tried the best using different task designs and analysis methods, including checking the quality of the data. Yet the null results remained. This means that, even if the difficulty effect existed, it was too weak for our experimental paradigm to detect it.

### 3.7. Our results contributed to better understanding on brain responses to task difficulty

In the introduction, we presented a prior fMRI study on orientation discrimination that showed that difficulty did not affect the activity across early visual areas including V1, V2, V3, and V4 [26]; and another study [27] showing a similar null effect of difficulty, when external noise was added to the stimuli. Now with our study drawing a similar conclusion with these previous studies, we can put the results together for a more comprehensive overview. First, our study measured the fMRI responses to motion stimuli, while Li et al. [26] studied orientation discrimination of gating stimuli. The task difficulty in the current study refers to the difficulty level manipulated by directional deviation in a motion direction discrimination task. Second, we investigated the responses in more brain areas than Li et al. [26]’s study. Beyond early visual areas, we examined mid-level and high-level areas such as MT+, IPS, and many spherical ROIs in higher-order cortices. Third, we measured fMRI responses in both the blocked and event-related designs. Lastly, we performed a series of systematic analyses as well as a supplementary experiment to verify the robustness of our analysis results and also investigate into the results with suggestive meanings. In summary, we believe that our results have contributed substantially to the understanding of brain responses as modulated by task difficulty.

## Supporting information

S1 Fig Behavioral results: Supplementary experiment. Behavioral accuracies averaged across 4 participants in 3°, 9°, 15°, and 80° conditions for the blocked design (A) and the event-related design (B).
(TIF)

S2 Fig MRI results of ROI analysis: Supplementary experiment. BOLD amplitudes averaged across 4 participants for the 3°, 9°, 15°, and 80° conditions in V1, V2, V3, V3a, V4, MT+, and IPS for the blocked design (A) and the event-related design (B).
(TIF)

S3 Fig Response time courses for the event-related design. Time courses of BOLD responses (measured as the signal percentage change) during 16.5 s after stimulus onset averaged across 14 (or 10 or 4) participants for three (or four) conditions in V1, V2, V3, V3a, V4, MT+, and IPS for the event-related design in Experiment 1 (A), Experiment 2 (B), and Supplementary Experiment (C).
(TIF)

S4 Fig MRI results of ROI analysis: Experiment 1 (selected participants only). BOLD amplitudes averaged across 4 selected participants for the 3°, 9°, and 15° conditions in V1, V2, V3, V3a, V4, MT+, and IPS for the blocked design (A) and the event-related design (B).
(TIF)

S5 Fig MRI results of ROI analysis: Experiment 2 (selected participants only). BOLD amplitudes averaged across 4 selected participants for the 3°, 9°, 15°, and 80° conditions in V1, V2, V3, V3a, V4, MT+, and IPS for the blocked design (A) and the event-related design (B).
(TIF)

S1 Text Methods. (DOCX)

S1 File Additional analyses and results. (DOCX)
