# Supplementary figures and images for: Effect of task difficulty on blood-oxygen-level-dependent signal: A functional magnetic resonance imaging study in a motion discrimination task
Source: PLoS One. 2018 Jun 25;13(6):e0199440. doi: 10.1371/journal.pone.0199440 (PMC6016936; doi:10.1371/journal.pone.0199440)

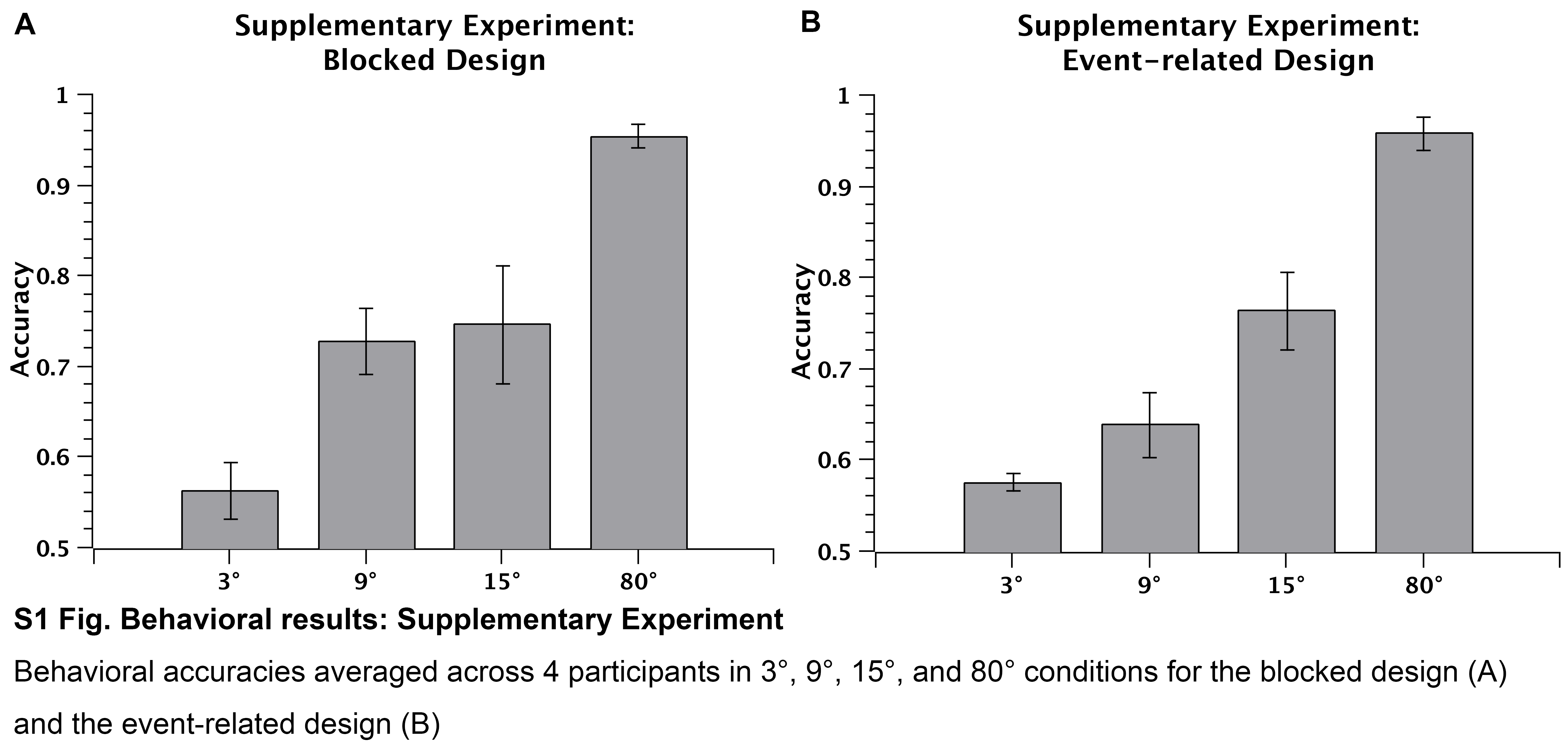

Supplement: S1 Fig — Behavioral accuracies averaged across 4 participants in 3°, 9°, 15°, and 80° conditions for the blocked design (A) and the event-related design (B). (TIF) [file pone.0199440.s001.tif]

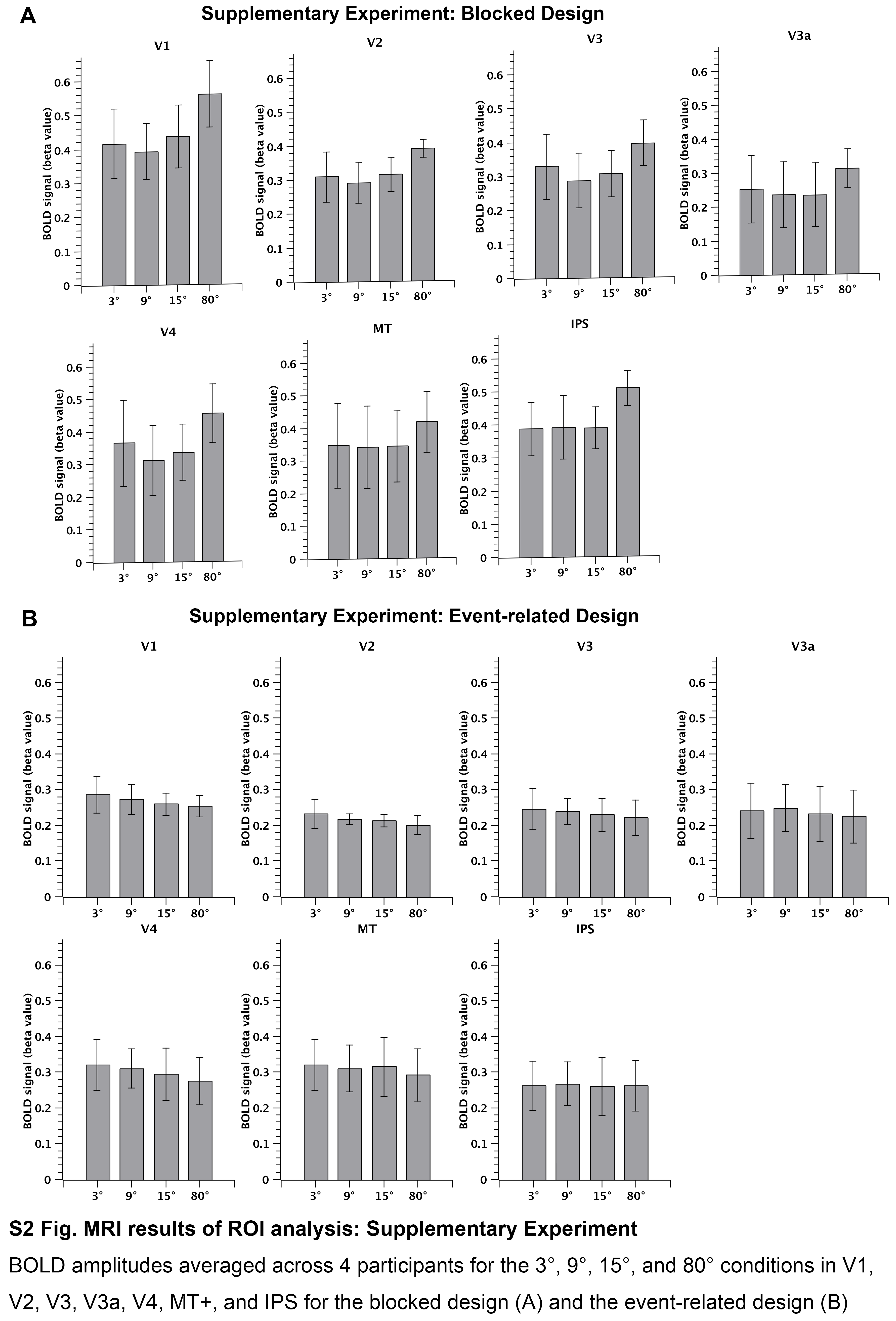

Supplement: S2 Fig — BOLD amplitudes averaged across 4 participants for the 3°, 9°, 15°, and 80° conditions in V1, V2, V3, V3a, V4, MT+, and IPS for the blocked design (A) and the event-related design (B). (TIF) [file pone.0199440.s002.tif]

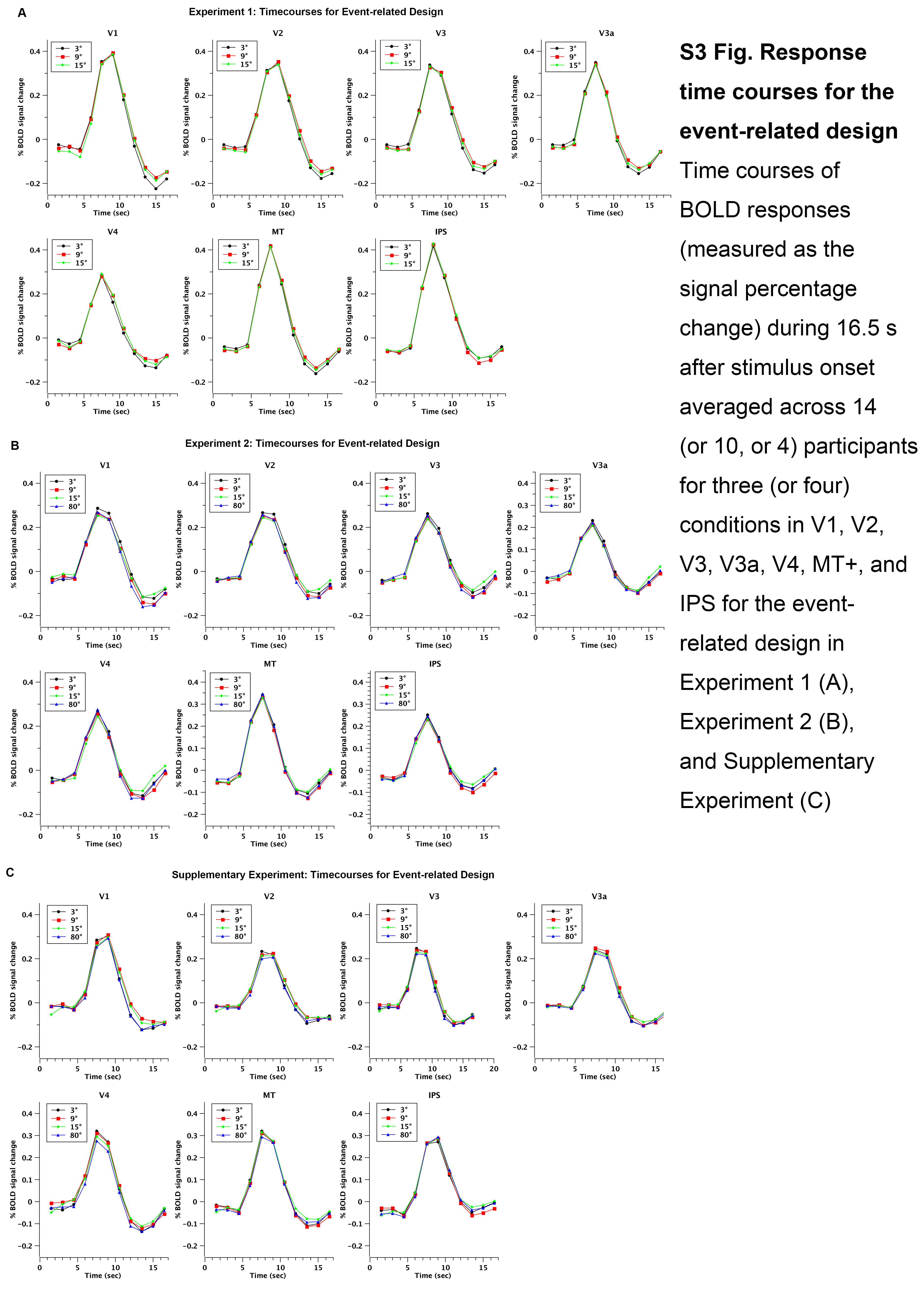

Supplement: S3 Fig — Time courses of BOLD responses (measured as the signal percentage change) during 16.5 s after stimulus onset averaged across 14 (or 10 or 4) participants for three (or four) conditions in V1, V2, V3, V3a, V4, MT+, and IPS for the event-related design in Experiment 1 (A), Experiment 2 (B), and Supplementary Experiment (C). (TIF) [file pone.0199440.s003.tif]

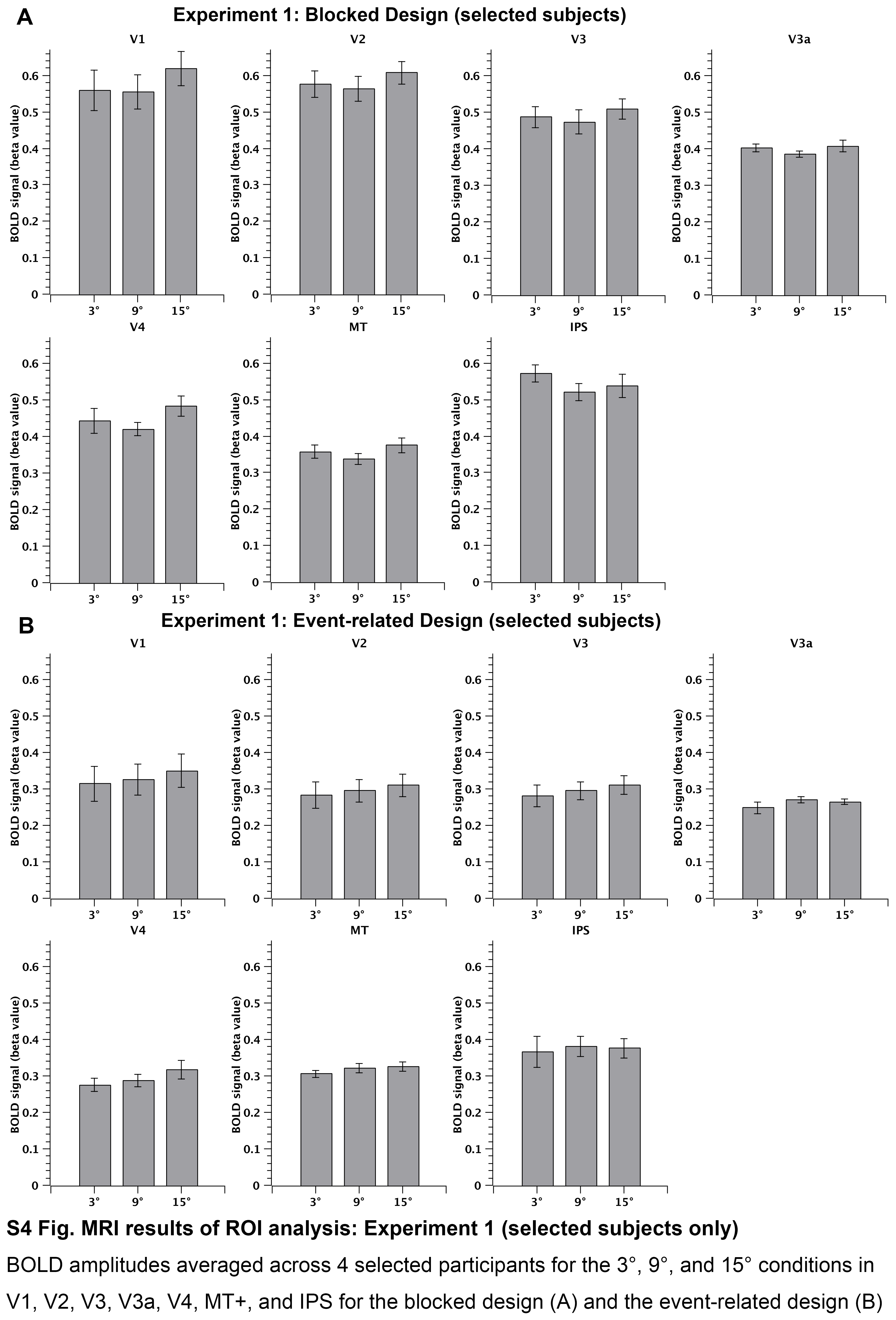

Supplement: S4 Fig — BOLD amplitudes averaged across 4 selected participants for the 3°, 9°, and 15° conditions in V1, V2, V3, V3a, V4, MT+, and IPS for the blocked design (A) and the event-related design (B). (TIF) [file pone.0199440.s004.tif]

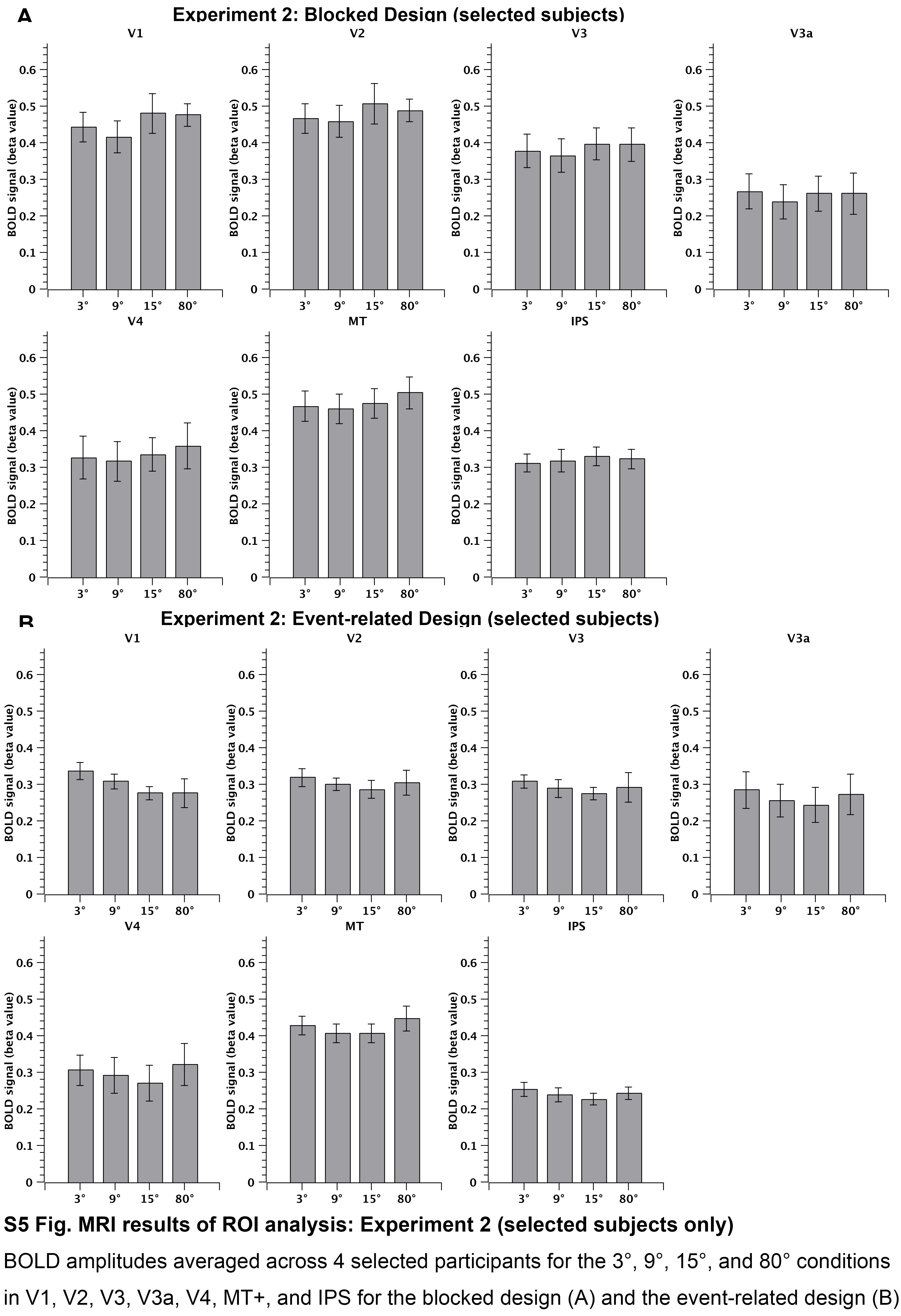

Supplement: S5 Fig — BOLD amplitudes averaged across 4 selected participants for the 3°, 9°, 15°, and 80° conditions in V1, V2, V3, V3a, V4, MT+, and IPS for the blocked design (A) and the event-related design (B). (TIF) [file pone.0199440.s005.tif]
