# Supplementary material for: Effect of task difficulty on blood-oxygen-level-dependent signal: A functional magnetic resonance imaging study in a motion discrimination task
Source: PLoS One. 2018 Jun 25;13(6):e0199440. doi: 10.1371/journal.pone.0199440 (PMC6016936; doi:10.1371/journal.pone.0199440)
Supplement: S1 Text — (DOCX) [file pone.0199440.s006.docx]

**Methods**

1. **Participants**

There were twenty-eight participants (14 for Experiment 1, 10 for Experiment 2, 4 for Supplementary Experiment; 10 males) in the study. All of them were right-handed, with reported normal or corrected-to-normal visual acuity, and had no known neurological or visual disorders. Their ages ranged from 20 to 25 years. All participants gave written, informed consent in accordance with the procedures and protocols approved by the human subject review committee of Peking University, Beijing, China.

1. **Stimuli and apparatus**

The reference stimulus was a cross with one of the four arms longer than the other three. The direction of the longest arm indicated the reference direction (Fig 1A). The length of the shorter arm was 0.1° in visual angle, and that of the longer arm was 0.3°. The mean luminance of the cross was 12.1 cd/m^2^ whilst that of the background was 42.3 cd/m^2^. In Experiments 1 and 2, the motion stimulus was a random-dot kinematogram (RDK) that moved with 100% coherence and a speed of 10°/sec. The luminance of these 400 dots was the same as the cross. The Michelson contrast between the dots and their background was 55.5%. The 400 dots were presented within an 8° diameter circular region at the center of the screen. In the psychophysical part of the study, the stimuli were presented on an IIYAMA HM204DT 22-in monitor, with a spatial resolution of 1024 × 768 and a refresh rate of 60 Hz. Participants viewed the stimuli from a distance of 60 cm. Their head position was stabilized using a chin and head rest. Participants were asked to fixate on a small white dot presented at the center of the monitor between consecutive trials and during RDK presentations. In the Supplementary Experiment, an annular RDK stimulus containing dots moving only in the peripheral visual field was presented. As shown in Fig 1B, the moving dots were restricted to an annular region with an outer radius of 8° and an inner radius of 4°. The stimuli were otherwise the same as in Experiments 1 and 2.

1. **Experimental designs**
   1. **Experiment 1**

Participants were instructed to perform a motion direction discrimination task that was manipulated at three difficulty levels during the fMRI scanning. The schematic description of a trial is shown in Fig 1A. In each 3 sec trial, a cross was first presented at the center of the screen for 600 ms, indicating the reference direction. This reference was then replaced by an RDK motion stimulus, which was presented for 200 ms. Afterwards, participants judged the direction of the RDK relative to the reference direction (clockwise or counter-clockwise). Participants had to make a response by a key press during a time window of 2200 ms. Their reaction time in each trial was also recorded.

The reference direction was randomly chosen from all the possible directions in 0-360°. The motion direction of RDK was 3°, 9°, or 15° deviated from it (referred hereafter as 3°, 9°, and 15°), corresponding to three levels of task difficulty. The deviation of the RDK relative to the reference was either clockwise or counter-clockwise, which was randomly chosen across trials.

Two fMRI experimental designs were adopted: the blocked and event-related designs. In the blocked design, each 192 sec run consisted of 6 stimulus blocks of 18 sec, and was sandwiched by 12 sec fixation blocks. Therefore, the first and last blocks were fixation blocks. Each stimulus block consisted of 6 trials, all of the same difficulty level (either 3°, 9°, or 15° angular difference between the reference and motion stimulus). For the event-related runs, there were four types of trials: 3°, 9°, 15°, and blank. In a blank trial, a 3 sec blank screen was presented with the center fixation dot only. The participants were asked to fixate on it, with no behavioral task. Each 213 sec event-related run consisted of a total of 64 trials, 16 for each type of trial, with a 9 sec blank at the beginning and a 12 sec blank at the end. The orders of four types of trials were counterbalanced in each run using the M-sequence [1]. Such sequences were pseudo-random, which has the advantage of being perfectly counterbalanced n trials back, so that each type of trial was preceded and followed equally often by all types of trials, including itself.

In the fMRI experiment, each participant completed a total of 10 blocked runs and 10 event-related runs of motion discrimination across three days. During the first day, each participant completed the localizer run, 2 blocked runs, and 2 event-related runs of the task. The second and third days consisted of 4 blocked runs and 4 event-related runs, respectively. Before the fMRI, each participant performed the task with 4 blocked and 4 event-related runs that were the same as those used in fMRI, in order to familiarize the participant with the stimuli and experimental procedure. There was no feedback in the behavioral test or fMRI scanning. The behavioral accuracy and reaction times were averaged across trials of each condition.

- 1. **Experiment 2**

In this experiment, in addition to the three difficulty levels adopted in Experiment 1, we had a fourth condition, in which the angular difference between the RDK stimulus and the reference was 80°, corresponding to an extremely easy condition. There were a total of 10 blocked runs and 10 event-related runs of the task for each participant. Each 264 sec run for the blocked design consisted of 8 stimulus blocks of 18 sec, which was sandwiched by 12 sec fixation blocks, except that the first block at the beginning and last block at the end of the run were 18 sec blank blocks. Each experimental block consisted of 6 trials, each containing stimuli from one of four difficulty levels; 3°, 9°, 15°, or 80° angular difference. For the event-related runs, there were five types of trials: 3°, 9°, 15°, 80°, and blank. Each 411 sec run consisted of 18 sec blank blocks at the beginning and the end, and 125 experimental trials, 25 for each angular difference.

- 1. **Supplementary Experiment**

The experimental design was the same as in Experiment 2, except the RDK stimuli were restricted to an annular region with an outer radius of 8° and an inner radius of 4°, as illustrated in Fig 1B.

1. **Mapping regions of interest**

For each participant, we identified regions of interest (ROIs) using standard retinotopic mapping procedures. Retinotopic visual areas (V1, V2, V3, V3a, and V4) were defined by a standard phase-encoded method [2, 3], in which participants viewed a rotating wedge and expanding ring stimuli that created traveling waves of neural activity in the visual cortex. MT+ was identified based on responses to stimuli that alternated in time between moving and stationary dots, as per conventional methods [4, 5]. Moving dots traveled back and forth (10°/sec) within the 8° diameter circular aperture, alternating direction once per second for 12 sec, and followed by a stationary dot field for 12 sec. Intraparietal sulcus (IPS) was also identified using the motion localizer, as contiguous clusters of motion selective voxels superior to the parietal-occipital junction within the IPS.

1. **MRI data acquisition**

In the scanner, the stimuli were back-projected via a video projector (refresh rate: 60 Hz; spatial resolution: 1024 × 768) onto a translucent screen placed inside the scanner bore. Participants viewed the stimuli through a mirror located above their eyes. The viewing distance was 83 cm. Functional MRI data were collected using a 3T Siemens Trio scanner with a 12-channel phase-array coil. BOLD signals were measured with an EPI sequence (TE: 30ms; TR: 1500ms; FOV: 160 × 160 mm^2^; matrix: 64 × 64; flip angle: 90°; slice thickness: 5 mm; gap: 0 mm; number of slices: 24; slice orientation: axial). The bottom slice was positioned at the bottom of the temporal lobe. A high-resolution 3D structural data set (3D MPRAGE; 1 × 1 × 1 mm^3^ resolution) was collected before the functional runs in all tests.

1. **MRI data analysis – ROI data analysis**

Data analyses were performed with Statistical Parametric Mapping 8 (SPM8) (http://www.fil.ion.ucl.ac.uk/spm/; Wellcome Department of Imaging Neuroscience, London, UK). The anatomical volume for each participant in the retinotopic mapping session was transformed into the anterior commissure-posterior commissure (AC-PC) space [6]. The cortical surface was extracted and then inflated using BrainVoyager QX. Functional volumes in all the sessions were preprocessed with steps including 3D motion correction, linear trend removal, and high-pass filtering (0.015 Hz) [7]. Head motion within each fMRI test was less than 3 mm for each participant. The functional volumes were then aligned to the anatomical volume obtained in the retinotopic mapping session and transformed into the AC-PC space. The first 6 sec of BOLD signals were discarded to minimize transient magnetic saturation effects.

For each participant, we used a general linear model (GLM) to select the most responsive voxels (across both hemispheres) that showed a stronger response to the motion stimuli than to a fixation dot across all fMRI test sessions (p < 0.001. Bonferroni corrected) in visual cortex (V1, V2, V3, V3a, V4, IPS, and MT+) for further analysis. The selected voxels were confirmed by the independent motion localizer scan described above. For the blocked runs, the BOLD time course signal in a run was first extracted by averaging the data from all the voxels within an ROI. Then, beta values for different task difficulty blocks (three in Experiment 1; four in Experiment 2 and Supplementary Experiment) were estimated with a GLM method. The BOLD amplitudes for each of the three (or four) conditions were computed as the beta values averaged across 10 runs. A repeated-measures ANOVA of beta values was performed with condition (Experiment 1: 3°, 9°, and 15°; Experiment 2: 3°, 9°, 15°, and 80°) in each ROI.

The event-related BOLD signals were calculated separately for each ROI in each participant and experiment, following the method used by Larsson, Landy (8), Liu, Larsson (9), Fang, Boyaci (10). For each MRI scan, the time course of MR signal intensity was first extracted by averaging the data across all the voxels within the pre-defined ROIs and then normalizing by the mean intensity across the scan. Event-related averages were then performed for each trial type (four in Experiment 1: 3°, 9°, 15°, and blank; five in Experiment 2 and Supplementary Experiment: 3°, 9°, 15°, 80°, and blank) by averaging 10 time points (15 sec) after the onset of the reference stimulus. The response to the blank was subtracted from the response to the test stimulus. The peak of the subtracted response, which turned out to be at the fifth TR after the stimulus onset, was used in each ROI as a measure of the response amplitude. A repeated-measures ANOVA of response amplitude was performed (Experiment 1: 3°, 9°, and 15°; Experiment 2: 3°, 9°, 15°, and 80°) in each ROI.

Besides ROI analysis in the visual cortex, we also investigated the task difficulty effect in higher-order brain areas. After reviewing previous studies on task difficulty and brain activity, we selected 42 talairach coordinates (Table 1) from the literature and adopted ROI analysis based on these positions. We identified the spherical ROI with a radius of 7 mm centered at each talairach coordinate. For each of the 42 ROIs, BOLD signals for the blocked and event-related designs were analyzed respectively in the same way as for the visual cortex ROIs.

1. **Correlation between the blocked and event-related designs**

In every ROI and for each participant, the beta values for the blocked design and the peak responses (the fifth TR after stimulus onset) for the event-related design were averaged across all the trials for every difficulty level condition, and used in the correlation analysis. The Pearson correlation was applied to the beta values and peak responses across all 14 participants, 3 conditions, and 49 ROIs in Experiment 1; and across 10 participants, 4 conditions, and 49 ROIs in Experiment 2. The 49 ROIs were the total of ROIs in the visual cortex (V1, V2, V3, V3a, V4, MT+, and IPS in AC-PC space) and in the higher-order cortex (42 spherical ROIs in talairach space).

1. **MRI data analysis – MVPA**

Multi-variate pattern analysis (MVPA) was performed on data from the blocked design, using a standard correlation analysis of spatial activity pattern, as described by Haxby (11). For each ROI and each block, beta values were estimated in every voxel. Therefore, 20 beta values were obtained for each condition in each voxel. Spatial patterns of beta values (i.e., spatial activity pattern) were then extracted for each condition. These coefficients were averaged across all possible block combinations in each difficulty level. Within each ROI, we computed the correlation coefficients between the spatial activity patterns evoked by the same condition in different blocks, which were termed within-condition correlation coefficients. We also computed the correlation coefficients between the spatial activity patterns evoked by different conditions, which were termed between-condition correlation coefficients. The correlation coefficients were Fisher-transformed: ½ ln[(1+r)/(1-r)] [12, 13]. For Experiment 1, there were totally three within-condition correlation coefficients (3°, 9°, 15°) and three between-condition correlation coefficients (3° vs. 9°, 3° vs. 15°, 9° vs. 15°) for each participant in each ROI. For Experiment 2, the numbers of within-condition and between-condition correlation coefficients were 4 and 6 respectively. Next, we averaged all within-condition correlation coefficients and between-condition correlation coefficients for each participant in each ROI, then compared the two groups of correlation coefficients (Bonferroni corrected by ROI). The correlation coefficients within each condition and between all condition combinations from Experiment 1 are shown in Fig 4A. The average correlation coefficients from Experiment 2 are shown in Fig 4B.

1. **MRI data analysis – whole brain search analysis**

A whole brain search analysis was performed on the data from the 3°, 9°, and 15° conditions of both the blocked and event-related data in Experiment 1 and 2, to see if any difficulty effects would be found elsewhere in the brain. Data were first preprocessed in SPM8 with head motion correction and realignment (the same as the method of ROI analysis). Then the images were spatially normalized to the standard Montreal Neurological Institute (MNI) Echo Planar Imaging (EPI) template, and spatially smoothed using a Gaussian kernel with a full width at half maximum of 8 mm. Intensity normalization and high-pass temporal filtering (using a filter width of 128 sec) were also applied to the data. After preprocessing, two levels of search analysis in MNI space were performed: 1) statistical analysis was conducted on individual data using GLM. The BOLD signal was modeled by convolving the design matrix with a canonical Hemodynamic Response Function (HRF). The following regressors were included in the design matrix: three conditions (3°, 9°, and 15°) and six scan-to-scan individual motion parameters produced during realignment. 2) The random effects group analysis was performed using contrasts of interest 3° vs. 9° and 9° vs. 15°. False discovery rate (FDR) correction was applied for multiple comparison correction.

1. **Correlation between behavioral and neural responses**

In order to investigate whether or not behavioral accuracy and response times are related to neural activity, we performed a trial-by-trial analysis, correlating behavioral responses with neural activity. Since this was a trial-by-trial analysis, only data from the event-related design could be included. We first separated all the trials into “correct” versus “incorrect” according to the behavioral responses. The average neural activity in each of the 49 ROIs (7 ROIs in the visual cortex and 42 spherical ROIs in the higher-order cortex) during the correct versus incorrect trials were compared. Second, we computed the Pearson correlation between the behavioral response times and the average neural activation, trial-by-trial, across all the participants (10 in Experiment 1 and 14 in Experiment 2) in each of 49 ROIs separately. Fisher-transformation was applied to the correlation values.

**References**

1. Buračas GT, Boynton GM. Efficient design of event-related fMRI experiments using M-sequences. Neuroimage. 2002;16(3):801-13. doi: 10.1006/nimg.2002.1116.

2. Sereno MI, Dale AM, Reppas JB, Kwong KK, Belliveau JW, Brady TJ, et al. Borders of multiple visual areas in humans revealed by functional magnetic resonance imaging. Science. 1995;268(5212):889-93.

3. Engel SA, Glover GH, Wandell BA. Retinotopic organization in human visual cortex and the spatial precision of functional MRI. Cerebral cortex. 1997;7(2):181-92.

4. Tootell RBH, B. RJ, K. KK, Malach R, Born RT, Brady TJ, et al. Functional analysis of human MT and related visual cortical areas using magnetic resonance imaging. Journal of Neuroscience. 1995;15(4):3215.

5. Huk AC, Dougherty RF, Heeger DJ. Retinotopy and functional subdivision of human areas MT and MST. The Journal of Neuroscience. 2002;22(16):7195-205.

6. Talairach J, Tournoux P. Co-planar stereotaxic atlas of the human brain. 3-Dimensional proportional system: an approach to cerebral imaging. 1988.

7. Smith AM, Lewis BK, Ruttimann UE, Ye FQ, Sinnwell TM, Yang Y, et al. Investigation of low frequency drift in fMRI signal. Neuroimage. 1999;9(5):526-33.

8. Larsson J, Landy MS, Heeger DJ. Orientation-selective adaptation to first-and second-order patterns in human visual cortex. Journal of neurophysiology. 2006;95(2):862-81. doi: 10.1152/jn.00668.2005.

9. Liu T, Larsson J, Carrasco M. Feature-Based Attention Modulates Orientation-Selective Responses in Human Visual Cortex. Neuron. 2007;55(2):313-23. doi: 10.1016/j.neuron.2007.06.030.

10. Fang F, Boyaci H, Kersten D. Border ownership selectivity in human early visual cortex and its modulation by attention. The Journal of Neuroscience. 2009;29(2):460-5. doi: 10.1523/JNEUROSCI.4628-08.2009.

11. Haxby JV. Multivariate pattern analysis of fMRI: the early beginnings. Neuroimage. 2012;62(2):852-5. Epub 2012/03/20. doi: 10.1016/j.neuroimage.2012.03.016. PubMed PMID: 22425670; PubMed Central PMCID: PMCPMC3389290.

12. Fisher RA. Frequency distribution of the values of the correlation coefficient in samples from an indefinitely large population. Biometrika. 1915;10:507-21.

13. Fisher RA. On the probable error of a coefficient of correlation deduced from a small sample. Metron. 1921;1:3-32.
