# Supplementary material for: Effect of task difficulty on blood-oxygen-level-dependent signal: A functional magnetic resonance imaging study in a motion discrimination task
Source: PLoS One. 2018 Jun 25;13(6):e0199440. doi: 10.1371/journal.pone.0199440 (PMC6016936; doi:10.1371/journal.pone.0199440)
Supplement: S1 File — (DOCX) [file pone.0199440.s007.docx]

**Additional analyses and results**


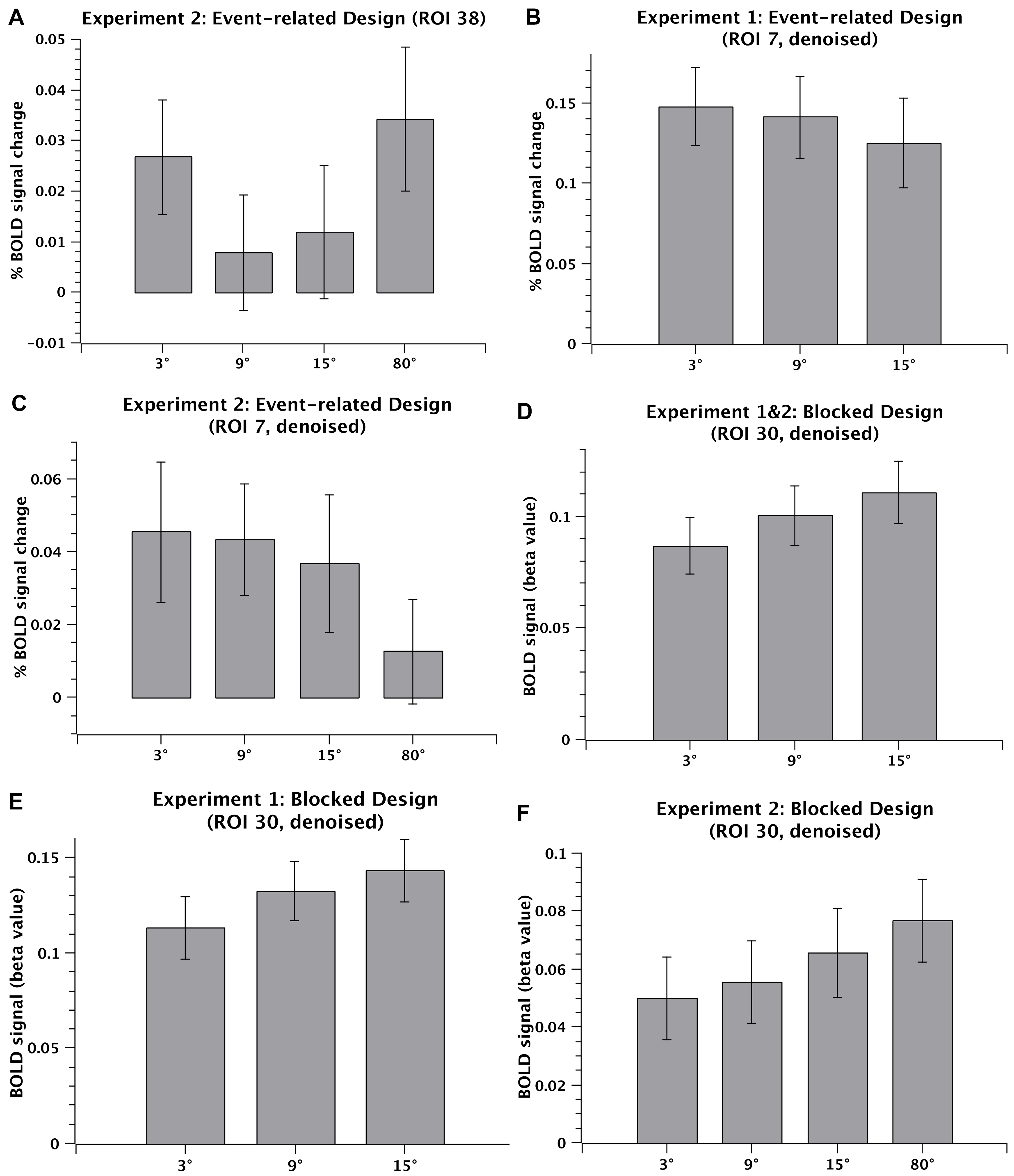


1. **Applying spatial smoothing to the data**

In the data preprocessing of Experiment 2, spatial smoothing was applied with an 8mm FWHM Gaussian kernel. The other analysis procedures were the same as the methods in S1 Text. The results showed that no significant difference across four conditions was found in any ROI after correction (p > 0.05 for the blocked design, and p > 0.05 for the event-related design, in 41 ROIs; p = 0.025 in 1 ROI, but with a non-monotonic function as the task difficulty decreased. Bonferroni corrected). This was consistent with the results using the non-smoothed data, and was further supportive of the conclusion that task difficulty did not affect neural activity in the brain. In the ROI reaching statistical significance in the middle frontal gyrus (ROI 38 in Fig A), the activation strength was very weak and changed in a non- monotonic fashion across the four conditions; therefore, we don’t believe there was a true task difficulty effect in this region.

1. **Comparison between 3° and 80° conditions**

In order to compare the easiest and the most difficult conditions directly, we calculated the difference of brain activations between the 3° and 80° conditions in Experiment 2. The results showed that no ROI exhibited a significant difference in brain activity between these two conditions (p > 0.05 for the blocked design; p > 0.05 for the event-related design. Bonferroni corrected).

1. **Applying denoising method to the data**

In order to improve the signal-to-noise ratio (SNRs) and the estimates of task-related components of the fMRI data, we applied Kendrick Kay’s denoising method [1] to the blocked and event-related data in both Experiments 1 and 2. After denoising, the averaged SNR values increased in both experiments for both designs. (Experiment 1, blocked design, from 3.44 to 3.80; event-related design, from 2.42 to 2.66. Experiment 2, blocked design, from1.86 to 2.25; event-related design from 1.56 to 1.89.) We applied the ROI analysis to the denoised data in the same way as in S4 Text. Our results showed that only one ROI in the anterior cingulate gyrus was significantly modulated by task difficulty for the event-related design in Experiment 2 (p = 0.025, Bonferroni corrected) (ROI 7 in Fig C). We also examined the activity in the same ROI for the event-related design in Experiment 1, but found a significant difference across the three conditions only before multiple comparison correction (p = 0.046, uncorrected) (Fig B).

In order to analyze data from both experiments, we combined the denoised data from the 14 participants in Experiment 1 and 10 participants in Experiment 2 and applied an ANOVA to all the 24 participants across the three difficulty conditions (3°, 9°, and 15°). The results showed that the activity of one ROI in the basal ganglia (ROI 30 in Fig D) was significantly different across the three conditions for the blocked design (p = 0.010, Bonferroni corrected). The activation strengths increased as the task became more difficult. When the same analysis was conducted at this ROI in Experiments 1 and 2 separately, marginal significance was obtained in both Experiment 1 (p = 0.078, Bonferroni corrected) (Fig E), and Experiment 2 (p = 0.094, Bonferroni corrected) (Fig F).

1. **Selecting the best participants for analysis**

We selected the best participants according to their behavioral data and imaging data, as assessed using a separate analysis. We took into consideration the behavioral accuracies, standard deviation of accuracy across runs, psychophysical tuning curves of behavioral data, head motion during fMRI scanning, signal-to-noise ratio of fMRI data, strength of BOLD signals, and variation of BOLD signals. Higher behavioral accuracies, smaller standard deviation of accuracies, smaller PSE (point of subject equality) and JND (just-noticeable difference) of the psychophysical curves, suggested better behavioral performance and the participants being more proactive, devoted, conscientious, and serious when performing the tasks. Smaller head motion, larger signal-to-noise ratios of fMRI data, larger strength and smaller variation of BOLD signals, suggested cleaner fMRI data with less noise interference. 4 out of 14 participants in Experiment 1 and 4 out of 10 participants in Experiment 2 were selected as the best participants according to the above criteria. The MRI results of Experiment 1 (S4 Fig) and Experiment 2 (S5 Fig) from the 4 selected best participants showed flat functions of BOLD response against task difficulty, which was consistent with the results from the whole participant group. This suggested that the participants’ subjective initiative or the fMRI data quality were not impeding us from finding significant task difficulty effects.

1. **Selecting better higher-order cortical ROIs for analysis**

In the main body of the article, we selected 42 higher-order cortical ROIs for analysis according to the existing results in previous articles concerning the task difficulty effect [2-6]. In order to focus more on the frontal and parietal areas with possible difficulty effects, we selected 25 Talairach coordinates in the frontal and parietal cortex out of the 42 ROIs for an additional analysis (Table 1). The ROI analysis was performed in the 25 spherical ROIs and none of them showed significant difference across task difficulty conditions (all p > 0.05, FDR corrected).

Next, among the articles we referred to, we selected Sunaert et al. [3]’s because the difficulty in their study was measured in a motion speed discrimination task, which was most close to the task paradigm in ours. The 6 Talairach coordinates in the frontal and parietal cortex from Sunaert et al. [3]’s article were picked out for an additional analysis (Table 2). The same ROI analysis was performed in these 6 spherical ROIs and the ones showing significant or marginally significant difference across difficulty conditions were demonstrated in the Figs G-J (In Experiment 1: for the blocked design *p* = 0.04 in ROI13, *p* = 0.04 in ROI17, *p* = 0.05 in ROI 19; for the event-related design *p* = 0.07 in ROI13. None of the ROIs was found for Experiment 2. FDR corrected). Nevertheless, when we performed an ANOVA to the combined data from the 14 participants in Experiment 1 and 10 participants in Experiment 2, these effects remained statistically significant for the blocked design (*p* < 0.05 in ROIs 13, 17, and 19. FDR corrected).


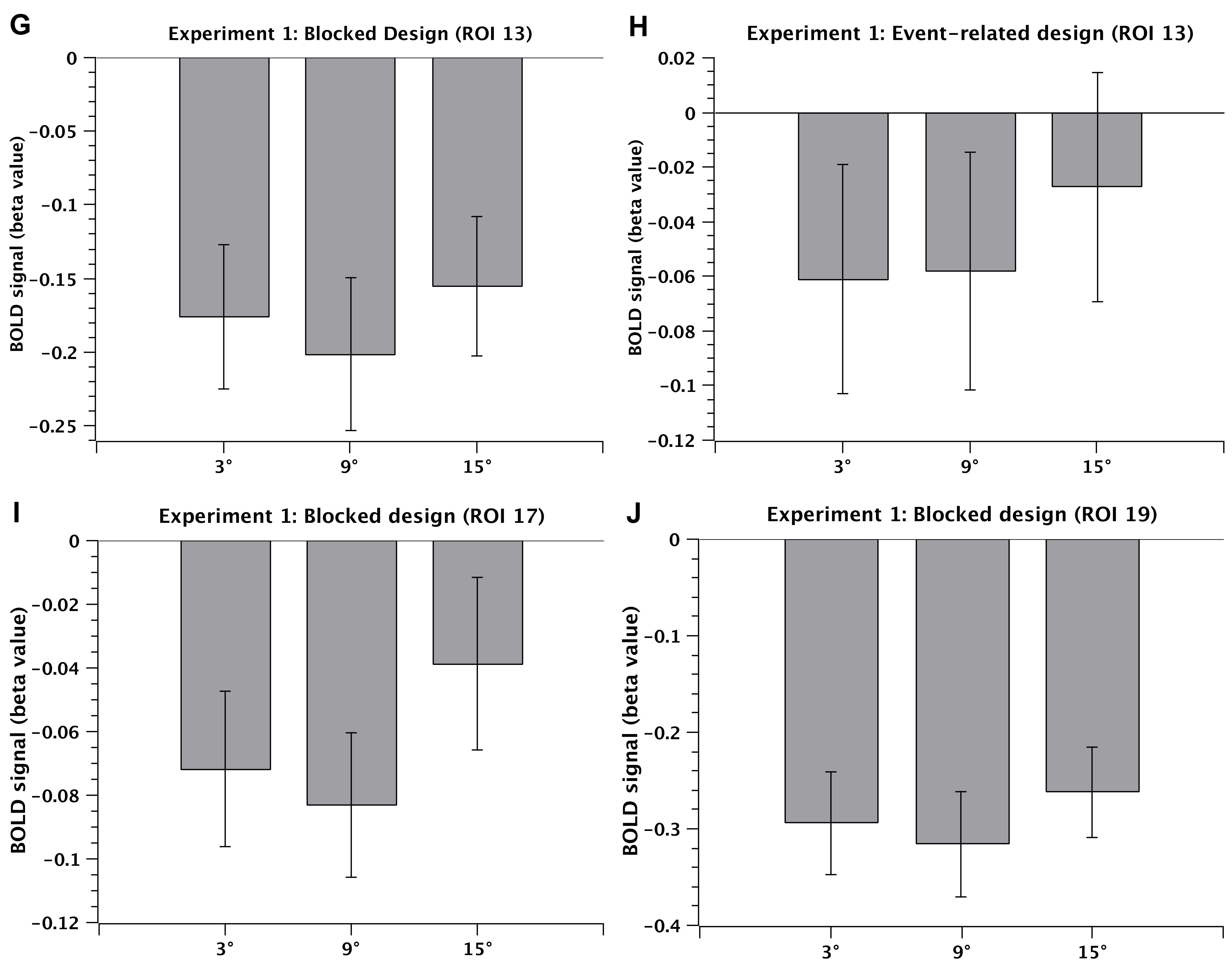


For Experiment 1, ROIs 13, 17, and 19, which were located at the superior and inferior frontal gyri, showed negative responses during task engagement, especially for the blocked design, implicating their possible involvement in the default mode network (DMN) activities. The individual difference in the structural organization of the DMN might result in the relatively large error bars. For Experiment 2, no statistically significant result was revealed. Nevertheless, when we performed an ANOVA to the combined data from the 14 participants in Experiment 1 and 10 participants in Experiment 2, these effects remained statistically significant for the blocked design (*p* < 0.05 in ROIs 13, 17, and 19, with FDR correction).

**References**

1. Kay KN, Rokem A, Winawer J, Dougherty RF, Wandell BA. GLMdenoise: a fast, automated technique for denoising task-based fMRI data. Front Neurosci. 2013; 7: 247.

2. Gould RL, Brown RG, Owen AM, ffytche DH, Howard RJ. FMRI BOLD response to increasing task difficulty during successful paired associates learning. NeuroImage. 2003; 20: 1006-1019.

3. Sunaert S, Van Hecke P, Marchal G, Orban GA. Attention to Speed of Motion, Speed Discrimination, and Task Difficulty: An fMRI Study. NeuroImage. 2000; 11: 612-623.

4. Dräger B, Jansen A, Bruchmann S, Förster AF, Pleger B, Zwitserlood P, et al. How does the brain accommodate to increased task difficulty in word finding? NeuroImage. 2004; 23: 1152-1160.

5. Barch DM, Braver TS, Nystrom LE, Forman SD, Noll DC, Cohen B. Dissociating working memory from task difficulty in human prefrontal cortex. Neuropsychologia. 1997; 35: 1373-1380.

6. Duncan J, Owen AM. Common regions of the human frontal lobe recruited by diverse cognitive demands. Trends Neurosci. 2000; 23: 475-483.

**Appendix**

**Table 1.**

| **ROI** | **Region** | **Hemisphere** | **Talairach coordinate** | | | **Reference** |
| --- | --- | --- | --- | --- | --- | --- |
|  |  |  | x | y | z |  |
| 1 | Precuneus | R | 12 | -65 | 51 | Gould et al. [2] |
| 3 | Precuneus | R | 4 | -58 | 53 |  |
| 13 | Superior frontal gyrus | L | -33 | 27 | 45 | Sunaert et al. [3] |
| 16 | Dorsal intraparietal sulcus | R | 27 | -60 | 51 |  |
| 17 | Inferior frontal gyrus | R | 45 | 21 | 15 |  |
| 18 | Inferior frontal gyrus | L | -51 | 30 | -3 |  |
| 19 | Superior frontal gyrus | L | -15 | 42 | 42 |  |
| 20 | Middle frontal gyrus | R | 39 | 45 | 24 |  |
| 23 | Superior parietal lobule | R | 27 | -63 | 54 | Dräger et al. [4] |
| 24 | Precuneous | R | 27 | -72 | 51 |  |
| 25 | Inferior parietal lobule | R | 42 | -51 | 60 |  |
| 26 | Postcentral gyrus | R | 45 | -39 | 57 |  |
| 27 | DLPFC | L | -37 | 42 | 29 | Barch et al. [5] |
| 28 | Inferior frontal cortex | L | -57 | 2 | 13 |  |
| 29 | Parietal cortex | L | -40 | -50 | 50 |  |
| 31 | Frontal lobe | R | 39 | 15 | 36 | Duncan, Owen [6] |
| 32 |  | R | 48 | 12 | 21 |  |
| 33 |  | L | -51 | 15 | 42 |  |
| 34 |  | L | 0 | 30 | 45 |  |
| 37 |  | R | 9 | 45 | -9 |  |
| 38 |  | R | 35 | 15 | 28 |  |
| 39 |  | R | 4 | 25 | 43 |  |
| 40 |  | R | 48 | 19 | 23 |  |
| 41 |  | R | 50 | 19 | 2 |  |
| 42 |  | R | 26 | 42 | 32 |  |

We selected 25 Talairach coordinates in the frontal and parietal cortex out of all the higher-order cortical ROIs for an additional analysis.

**Table 2.**

| **ROI** | **Region** | **Hemisphere** | **Talairach coordinate** | | | **Reference** |
| --- | --- | --- | --- | --- | --- | --- |
|  |  |  | x | y | z | Sunaert et al. [3] |
| 13 | Superior frontal gyrus | L | -33 | 27 | 45 |  |
| 16 | Dorsal intraparietal sulcus | R | 27 | -60 | 51 |  |
| 17 | Inferior frontal gyrus | R | 45 | 21 | 15 |  |
| 18 | Inferior frontal gyrus | L | -51 | 30 | -3 |  |
| 19 | Superior frontal gyrus | L | -15 | 42 | 42 |  |
| 20 | Middle frontal gyrus | R | 39 | 45 | 24 |  |

We selected 6 Talairach coordinates in the frontal and parietal cortex according to Sunaert et al. [3]’s article for an additional analysis.
